# Taxonomic revision of *Mordellistenahirtipes* species complex with new distribution records (Insecta, Coleoptera, Mordellidae)

**DOI:** 10.3897/zookeys.854.32299

**Published:** 2019-06-10

**Authors:** Dávid Selnekovič, Ján Kodada

**Affiliations:** 1 Department of Zoology, Faculty of Natural Sciences, Comenius University in Bratislava, Ilkovičova 6, SK-84215, Bratislava, Slovakia Comenius University Bratislava Slovakia

**Keywords:** taxonomy, identification key, distribution, new synonym, morphometry, Principal Component Analysis (PCA)

## Abstract

A taxonomic revision of species related to *Mordellistenahirtipes* Schilsky, 1895 is presented. Five species among the *M.hirtipes* complex are recognised: *M.hirtipes* Schilsky, 1895, *M.pseudohirtipes* Ermisch, 1965, *M.purpurascens* Costa, 1854, *M.balearica* Compte, 1985, and *M.irritans* Franciscolo, 1991. Descriptions, differential diagnoses, an identification key, and new distributional records are provided. Principal Component Analysis is performed for visualisation of differentiation between taxa. The following taxonomic acts are proposed: *Mordellistenapodlussanyi* Czető, 1990 and *M.aegea* Franciscolo, 1949 are proposed as junior subjective synonyms of *M.hirtipes* Schilsky, 1895; *M.fageli* Ermisch, 1969 and *M.pseudohirtipeskrotosensis* Czető, 1990 are proposed as junior subjective synonyms of *M.pseudohirtipespseudohirtipes* Ermisch, 1965; *M.geronensis* Ermisch, 1977 and *M.istrica* Ermisch, 1977 are proposed as junior subjective synonyms of *M.purpurascens* Costa, 1854.

## Introduction

*Mordellistena* Costa, 1854 is the largest genus within the family Mordellidae Latreille, 1802, comprising approximately 800 described species (Horák 2011). Adults are commonly found on flowers where they feed on pollen and nectar. Larvae are found in the stems of herbaceous plants or in decaying wood.

The Western Palaearctic species are conventionally assigned to species groups proposed by Ermisch (1956, 1969b), based on combinations of morphological characters. There are no studies focused on the phylogeny of Palaearctic species of genus *Mordellistena*, and thus the phylogenetic relationships among its species remain unresolved.

The present study is focused on nine morphologically related taxa, which represent a complex within *M.confinis* species group (Ermisch 1956, 1969b, Batten 1977). The unique character shared by all species of the complex is the expanded second segment of maxillary palpi in males, bearing very long setae on the ventral surface (Figs 4A, 6C, 7C). Additional characters are a strongly convex body and unique shape of parameres (Figs 5D–G, 6E–H, 7G–J, 8, 9).

Species belonging to this complex were described by Costa (1854), Schilsky (1895), Ermisch (1965, 1969a, 1977), Compte (1985), and Franciscolo (1949, 1991). Most of descriptions appeared to be insufficient for proper identification and after examination of type material, it became clear that some of the taxa are conspecific. In the present paper, we provide redescriptions of *M.hirtipes* Schilsky, 1895, *M.pseudohirtipes* Ermisch, 1965, and *M.purpurascens* Costa, 1854. Important diagnostic characters are visualised in drawings and photographs. We also performed Principal Component Analysis (PCA) based on morphometric measurements. This method is widely used in taxonomical research of invertebrates to help separate putative species in difficult species-complexes, to visualise differentiation between species and to evaluate the importance of peculiar morphometric characters (Kucharczyk et al. 2012, Przybycień and Wacławik 2015).

Examination of material from several localities in Western Palaearctic revealed new distribution records and new biological information for *M.hirtipes* Schilsky, 1895, *M.pseudohirtipes* Ermisch, 1965, and *M.purpurascens* Costa, 1854.

## Materials and methods

Dried specimens were relaxed in water with a few drops of acetic acid to allow for the dissection. Specimens were observed using Leica MZ16 stereomicroscope with magnification up to 120×, illuminated with diffuse light (neon bulb, 6400 K). Dissected body parts for drawings were temporarily mounted on slides in glycerine. Drawings were prepared using Leica drawing tube attached to Leica DM 1000 microscope, scanned and traced in Adobe Illustrator CS6. Dissected body parts were after examination mounted on the same card as respective specimen using dimethyl hydantoin formaldehyde (DMHF) or put to genitalia microvials filled with glycerine and pinned with the respective specimen. Measurements were taken using ocular micrometre. Intervals of measured values are followed by data in parentheses: arithmetic mean ± standard deviation, n = number of measured specimens. Total length (TL) was measured from the anterior margin of pronotum to the apices of elytra; elytral length (EL) was measured from the apex of scutellar shield to the apices of the elytra; elytral width (EW) was measured at the widest point of elytra. Digital photographs were taken using Canon EOS 5D mark II camera attached to Zeiss Axio Zoom.V16 stereomicroscope. Image stacks were produced manually, combined using Zerene Stacker software and edited in Adobe Photoshop CC.

Terminology used in morphological descriptions follows Lawrence and Ślipiński (2010) and is supplemented by terminology used by Lu et al. (1997) for the genitalia.

Specimen data are given in the following format: number of specimens and sex, depository: exact data from labels in quotation marks; slash indicates separate labels; author’s remarks are given in square brackets.

Principal Component Analysis (PCA) was conducted in PAST 3.12 software (Hammer et al. 2001), based on variance-covariance matrix of 12 morphometric characters: HL, HW, PL, PW, EL, EW, PTiL, MsTiL, MtTiL, RPrL, BRPr, and LPrL. Measurements were taken from 59 male specimens (including holotypes / lectotypes) assigned to eight taxa (*M.hirtipes* Schilsky, 1895, *M.podlussanyi* Czető, 1990, *M.aegea* Franciscolo, 1949, *M.pseudohirtipespseudohirtipes* Ermisch, 1965, *M.pseudohirtipeskrotosensis* Czető, 1990, *M.fageli* Ermisch, 1969, *M.geronensis* Ermisch, 1977, and *M.istrica* Ermisch, 1977). Lectotype of *M.purpurascens* was not measured because of its bad condition. Plot created in PAST 3.12 was subsequently edited in Adobe Illustrator CS6.

All nomenclatorial acts follow regulations of ICZN (1999).

Overall 149 specimens from following depositories were examined:

**CSB** collection of Dávid Selnekovič, Bratislava, Slovakia

**HNHM** Hungarian Natural History Museum, Budapest, Hungary

**MCST** Museo Civico di Storia Naturale, Trieste, Italy

**MNCN** Museo Nacional de Ciencias Naturales, Madrid, Spain

**MNHU** Museum für Naturkunde der Humboldt Universität, Berlin, Germany

**MZFN** Museo Zoologico dell’Università Federico II, Naples, Italy

**SNSD** Senckenberg Naturhistorische Sammlungen, Dresden, Germany

Abbreviations of measured characters:

**BLPr** basal part of left paramere length

**BRPr** basal part of right paramere length

**EL** length of elytra

**EW** width of elytra (combined)

**HL** length of head

**HW** width of head

**LabL** length of labrum

**LabW** width of labrum

**LPrL** length of left paramere

**MsTiL** length of mesotibiae

**MsTrL** length of mesotarsi

**MtTiL** length of metatibiae

**MtTrL** length of metatarsi

**PL** length of pronotum

**PTiL** length of protibiae

**PTrL** length of protarsi

**PW** width of pronotum

**PygL** length of pygidium

**RPrL** length of right paramere

**St8L** length of sternite VIII

**St8W** width of sternite VIII

**TVtL** length of terminal abdominal ventrite

**TL** total length

**TPalL** length of terminal segment of maxillary palpi

**TPalW** width of terminal segment of maxillary palpi.

### Data resources

The data underpinning the analyses reported in this paper are deposited at GBIF, the Global Biodiversity Information Facility, https://doi.org/10.15468/pkhkul

## Taxonomy

### *Mordellistena* Costa, 1854

*Mordellistena* Costa, 1854: 16 [type species: *Mordellistenaconfinis* Costa, 1854: 18]

#### Mordellistena hirtipes

Taxon classificationAnimaliaColeopteraMordellidae

species complex

##### Diagnosis.

Integument including legs and maxillary palpi completely black; metatibial spurs black; pubescence of dorsum yellowish, sometimes darkened in apical portions of elytra but never completely dark. Antennomeres I–IV shorter and narrower than following ones (Figs 5A, B, 6A, B, 7A, B). Maxillary palpomere II expanded in males, bearing very long setae on ventral surface (Figs 4A, 6C, 7C). Metatibiae at least with three lateral ridges, all parallel to apical margin of tibia. Metatarsomeres I and II with ridges.

#### Mordellistena (s. str.) hirtipes

Taxon classificationAnimaliaColeopteraMordellidae

Schilsky, 1895

**Figures 1–3.**
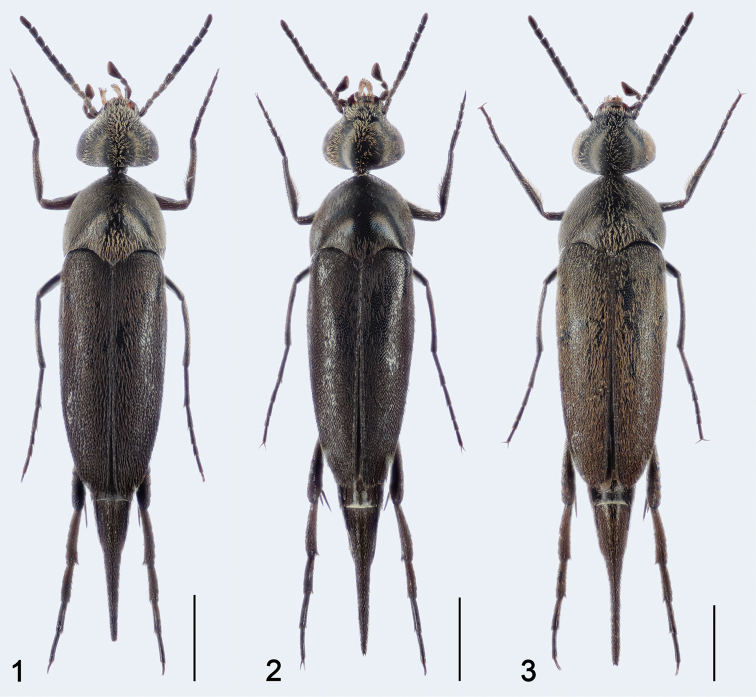
**1***Mordellistenahirtipes* Schilsky, 1895, male **2***M.pseudohirtipes* Ermisch, 1965, male **3***M.geronensis* Ermisch, 1977, male. Scale bar: 0.50 mm.

**Figure 4.**
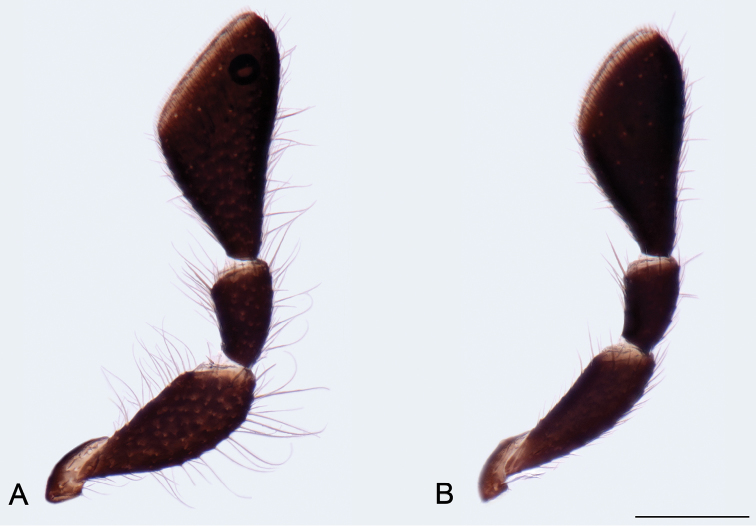
*Mordellistenahirtipes* Schilsky, 1895, maxillary palpi: **A** male **B** female. Scale bar 0.10 mm.

**Figure 5.**
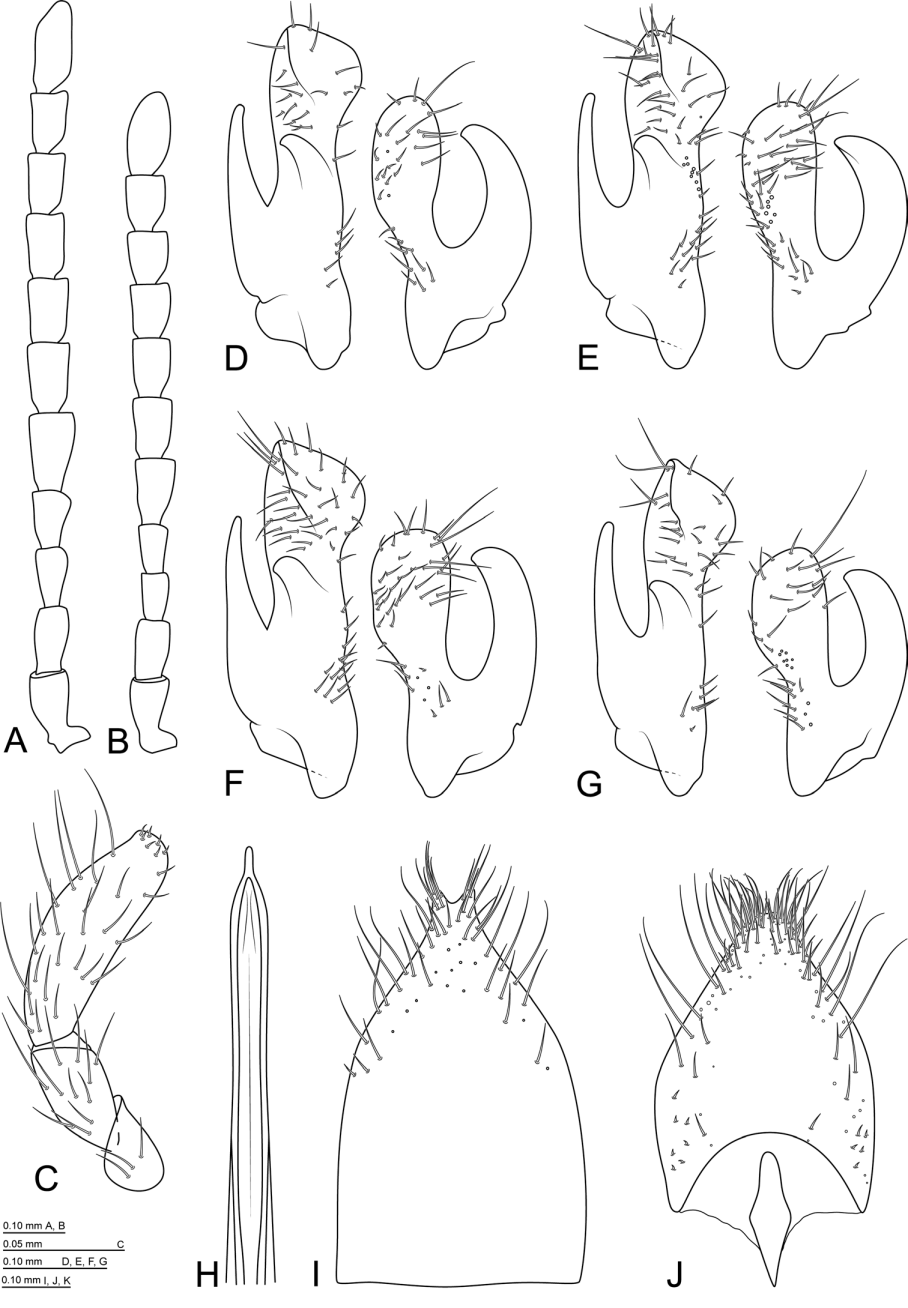
*Mordellistenahirtipes* Schilsky, 1895: **A** antenna, male **B** antenna, female **C** labial palpus, female **D** parameres, lectotype **E** parameres, holotype of *M.podlussanyi***F** parameres, Greece **G** parameres, Spain **H** aedeagal median lobe **I** sternite VIII, male **J** sternite VIII, female.

Figs 1 , 4A, B , 5A–J

Mordellistena hirtipes Schilsky, 1895: 46 (original description); Heyden et al. 1906: 455 (catalogue); Csiki 1915: 35 (catalogue); Schaufuss 1916: 766 (distribution); Ermisch 1963: 62 (distribution); Ermisch 1965: 268 (distribution); Batten 1976: 168 (distribution); Batten 1977: 171–173 (distribution, figures, key); Horák 1990: 136 (lectotype and paralectotypes designation, figures); Odnosum 2003: 36–37, 40, 46 (key, figures, distribution); Horák 2008: 98 (catalogue, distribution); Odnosum 2010: 153, 192–194 (key, description, figures, distribution); Samin et al. 2016: 24 (distribution); Ruzzier et al. 2017: 152 (distribution).
Mordellistena aegea Franciscolo, 1949: 90, 93 syn. nov. (original description); Batten 1977: 169 (remarks); Horák 2008: 96 (catalogue).
Mordellistena podlussanyi Czető, 1990: 26–29 syn. nov. (original description); Horák 2008: 100 (catalogue).

##### Type locality.

Attalia [Turkey].

##### Type material examined.

*M.hirtipes*: LECTOTYPE (by designation of Horák (1990: 136)): 1 ♂, MNHU: “Attalia Reitter [hand written] / hirtipes Schils. [hand written] / Type [red label] / Zool. Mus. Berlin / [card with dissected genitalia] / LECTOTYPUS [red label] / Mordellistenahirtipes Schils. J. Horák det. 1985”; PARALECTOTYPES (by designation of Horák (1990: 136)): 4 ♂♂, 3 ♀♀, MNHU: “Attalia Reitter [hand written] / Coll. Schilsky / Type [red label] / Zool. Mus. Berlin / PARALECTOTYPUS [red label] / Mordellistenahirtipes Schils. J. Horák det. 1985”; 17 ♂♂, 9 ♀♀, MNHU: “♂ [or] ♀ / Coll. Schilsky / Type [red label] / Zool. Mus. Berlin / PARALECTOTYPUS [red label] / Mordellistenahirtipes Schils. J. Horák det. 1985”; 1 ♀, MNHU: “Syrien Kaifa. Reitter. / Coll. Schilsky / Type [red label] / Zool. Mus. Berlin / PARALECTOTYPUS [red label] / Mordellistenahirtipes Schils. J. Horák det. 1985”; 1 ♀, MNHU: “Morea Hagios Wlassis Brenske / hirtipes [hand written] / Type [red label] / Zool. Mus. Berlin / PARALECTOTYPUS [red label] / Mordellistenahirtipes Schils. J. Horák det. 1985”. *M.aegea*: HOLOTYPE: 1 ♂, MCST: “Pod. Sper. Coo 7. VII.–VIII. [Podere sperimentale, Kos Island; hand written] / 7. VII.–VIII. Pod. Sper. Coo [hand written] / 19 [blue label] / [card with dissected median lobe] / Olo [hand written] Typus / [cover slides with dissected parameres and sternite VIII] / Mordellistenaaegea n. sp. DET. FRANCSCOLO / HOLOTYPUS Mordellistenaaegea Franciscolo, 1949 D. Selnekovič labelled 2018 / Mordellistena (s. str.) hirtipes Schilsky, 1895 D. Selnekovič det. 2018”. *M.podlussanyi*: HOLOTYPE: 1 ♂, HNHM: “♂ / [transparent plastic board with dissected genitalia] / Krotos KRÉTA / 1981. V. 12. leg. Podlussány / Holotypus Mordellistenapodlussanyi Czető, 1988 [red label, hand written] / Mordellistena (s. str.) hirtipes Schilsky, 1895 D. Selnekovič det. 2017”.

##### Additional material examined.

**Croatia**: 1 ♂, HNHM: “Dalmatia leg. Endrödy-Younga / Dubrovnik Ins. Lokrum / 1958. VIII. 7. Kätscher / Mordellistenahirtipes Schils. det. R. Batten 1979 / Mordellistena (s. str.) hirtipes Schilsky, 1895 D. Selnekovič det. 2017”. **Cyprus**: 1 ♂, HNHM: “Cyprus Laranka Glaszner / Mordellistenahirtipes Schils. det. R. Batten 1979 / Mordellistena (s. str.) hirtipes Schilsky, 1895 D. Selnekovič det. 2017”; 12 ♂♂, 2 ♀♀, CSB: “Cyprus W, Limassol env., Germasogeia Reservoir 34°45'19"N, 33°05'36"E, 27. IV. 2018 D. Selnekovič leg. / Mordellistena (s. str.) hirtipes Schilsky, 1895 D. Selnekovič det. 2018”. **Greece**: 1 ♂, SNSD: “♂ / Insel Rhodos 24.5.–5.8.62 / Stadt Rhodos Umg. Dr Mand / Genitalpräparat / MORDELLISTENA pseudohirtipes Erm. K. Ermisch det. 19 / Mordellistena (s. str.) hirtipes Schilsky, 1895 D. Selnekovič det. 2017”; 1 ♂, HNHM: “Crete Biró / Ins. Dia 25.–29. V. / Mordellistenahirtipes Schils. det. R. Batten 1980 / Mordellistena (s. str.) hirtipes Schilsky, 1895 D. Selnekovič det. 2017”. **Montenegro**: 3 ♂♂, 3 ♀♀, CSB: “Montenegro SW Bar city env. 42°07'56"N, 19°07'33"E, 22. VI. 2011 D. Selnekovič / Mordellistena (s. str.) hirtipes Schilsky, 1895 D. Selnekovič det. 2012”. 2 ♂♂, 3 ♀♀, CSB: “Montenegro SW Bar city–Volujica hill 242°04'16"N, 19°06'10"E, 20. VI. 2011 D. Selnekovič / Mordellistena (s. str.) hirtipes Schilsky, 1895 D. Selnekovič det. 2012”. 2 ♂♂, 2 ♀♀, CSB: “Montenegro SW Bar city–Stari Bar 42°05'31"N, 19°07'58"E, D. Selnekovič 19. VI. 2011 / Mordellistena (s. str.) hirtipes Schilsky, 1895 D. Selnekovič det. 2012”. 1 ♂, CSB: “Montenegro SW Bar city, on Daucus42°06'N, 19°06'E, 19. VI. 2011 D. Selnekovič 2011 / Mordellistena (s. str.) hirtipes Schilsky, 1895 D. Selnekovič det. 2012”. **Spain**: 1 ♂, 1 ♂, SNSD: “Spanien, Prov. Gerona Tossa de mar A. Kampf, VII–VIII 35 / Paratypus Mordellistenageronensis Ermisch / Mordellistena (s. str.) hirtipes Schilsky, 1895 D. Selnekovič det. 2017”. 1 ♂, SNSD: “Nordostspanien Costa brava 27. 7. 53 Dr David / Paratypus / PARATYPUS Mordellistena (s. str.) geronensis Ermisch, 1977 Selnekovič labelled 2017 [red label] / Mordellistena (s. str.) hirtipes Schilsky, 1895 D. Selnekovič det. 2017”.

##### Differential diagnosis.

**Figure 6.**
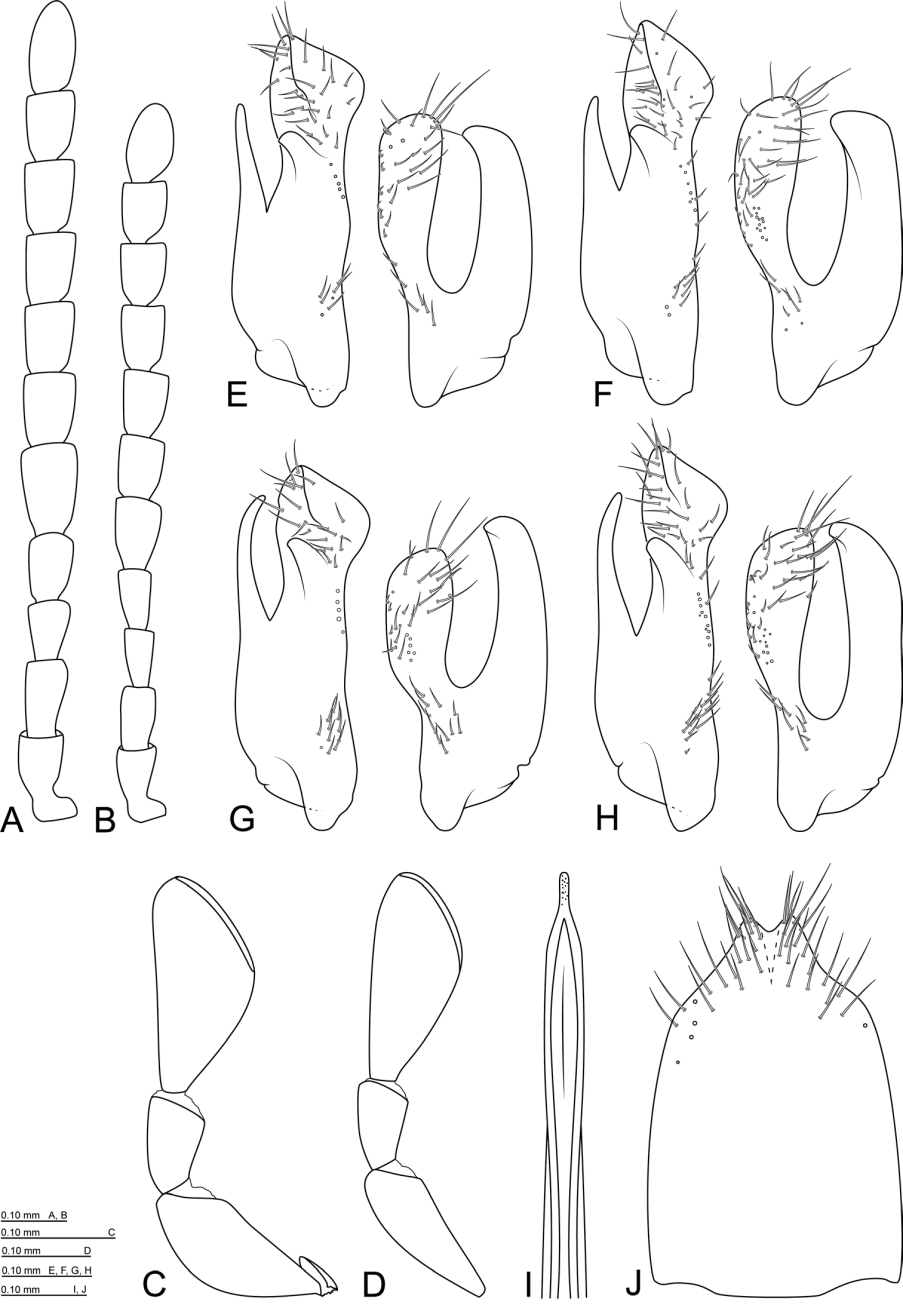
*Mordellistenapseudohirtipes* Ermisch, 1965: **A** antenna, male **B** antenna, female **C** maxillary palpus, male **D** maxillary palpus, female **E** parameres, holotype **F** parameres, holotype of M.pseudohirtipesssp.krotosensis**G** parameres, holotype of *M.fageli***H** parameres, France **I** aedeagal median lobe **J** sternite VIII, male.

**Figure 7.**
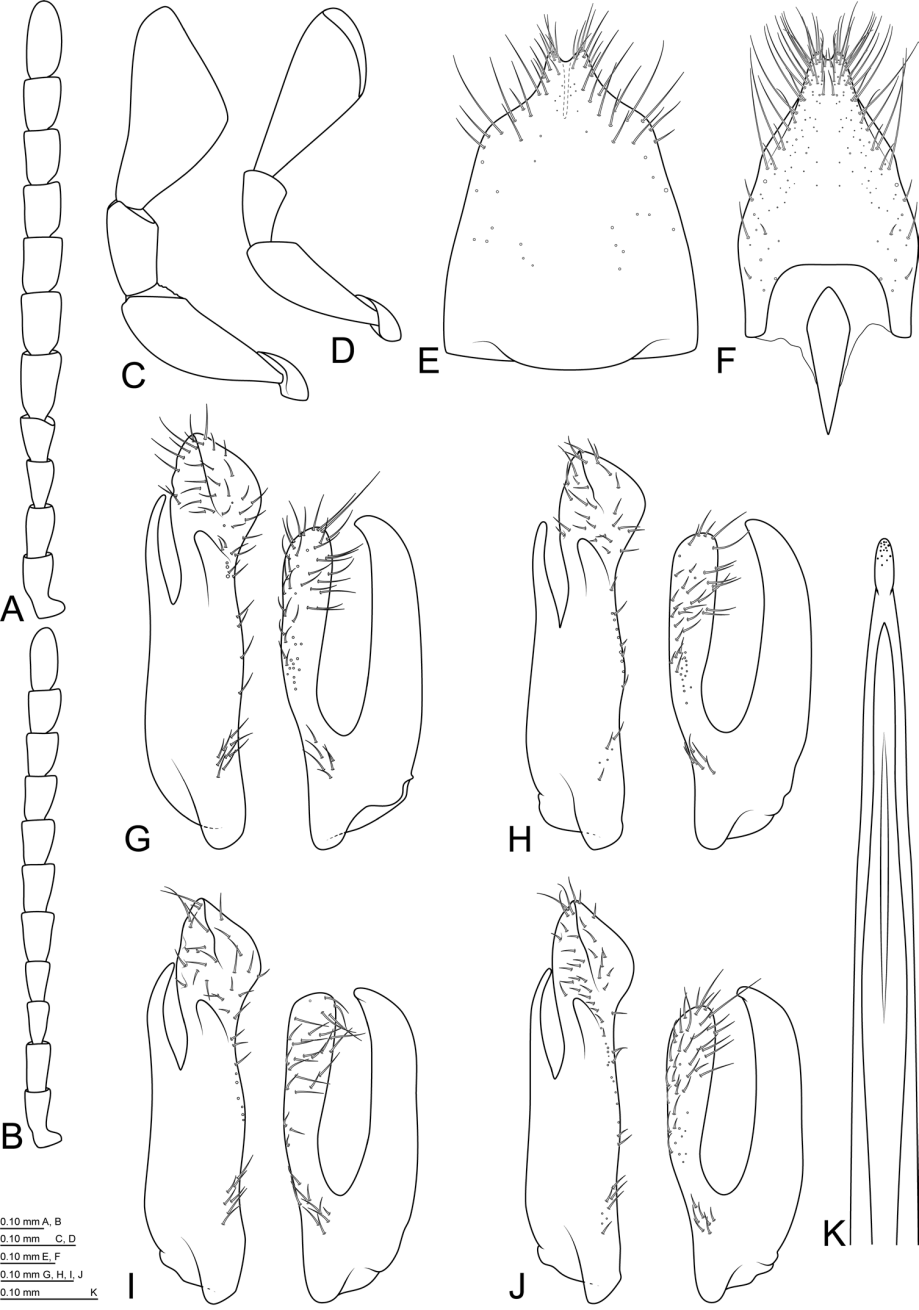
*Mordellistenapurpurascens* Costa, 1845: **A** antenna, male **B** antenna, female **C** maxillary palpus, male **D** maxillary palpus, female **E** sternite VIII, male **F** sternite VIII, female **G** parameres, lectotype **H** parameres, holotype of *M.geronensis***I** parameres, holotype of *M.istrica***J** parameres, France **K** aedeagal median lobe, holotype.

Parameres of *M.hirtipes* are shorter in proportion to the body dimensions than in *M.pseudohirtipes* and *M.purpurascens* (EL/LPrL ratio in *M.hirtipes*: 7.87–9.17 (8.48 ± 0.40, n = 14), *M.pseudohirtipes*: 4.65–7.17 (5.89 ± 0.71, n = 25) and *M.purpurascens*: 4.42–5.84 (4.98 ± 0.35, n = 19); EL/RPrL ratio in *M.hirtipes*: 10.07–11.89 (11.10 ± 0.50, n = 14), *M.pseudohirtipes*: 5.91–8.63 (7.42 ± 0.72, n = 25) and *M.purpurascens*: 5.57–6.94 (6.19 ± 0.41 n = 19). Ventral branch of the right paramere is in *M.hirtipes* (Fig. 5D–G) usually distinctly shorter than the dorsal one whereas in *M.pseudohirtipes* (Fig. 6E–H) and *M.purpurascens* (Fig. 7G–J) it is subequal or longer. Basal part of the left paramere is in *M.hirtipes* (Fig. 5D–G) distinctly shorter than in *M.purpurascens* (Fig. 7G–J). Sides of elytra less convergent apically than in *M.pseudohirtipes* and *M.purpurascens*. Terminal maxillary palpomere in females shorter and broader, its inner angle is more acute (Fig. 4B) than in *M.pseudohirtipes* (Fig. 6D) and *M.purpurascens* (Fig. 7D).

##### Redescription.

Measurements: TL: ♂♂ 3.21–3.95 mm (3.51 ± 0.24 mm, n = 13), ♀♀ 2.79–4.68 mm (3.44 ± 0.52 mm, n = 9); HL: ♂♂ 0.72–0.93 mm (0.81 ± 0.06 mm, n = 14), ♀♀ 0.67–0.80 mm (0.74 ± 0.04 mm, n = 9); HW: ♂♂ 0.87–1.02 mm (0.94 ± 0.05 mm, n = 14), ♀♀ 0.77–0.96 mm (0.87 ± 0.06 mm, n = 9); PL: ♂♂ 1.04–1.33 mm (1.17 ± 0.11 mm, n = 14), ♀♀ 0.94–1.20 mm (1.09 ± 0.08 mm, n = 9); PW: ♂♂ 1.06–1.37 mm (1.23 ± 0.10 mm, n = 14), ♀♀ 0.98–1.29 mm (1.16 ± 0.10 mm, n = 9); EL: ♂♂ 2.34–2.96 mm (2.65 ± 0.19 mm, n = 14), ♀♀ 2.08–2.79 mm (2.48 ± 0.21 mm, n = 9); EW: ♂♂ 1.10–1.43 mm (1.25 ± 0.11 mm, n = 14), ♀♀ 1.10–1.36 mm (1.23 ± 0.09 mm, n = 9); PTiL: ♂♂ 0.70–0.87 mm (0.77 ± 0.05 mm, n = 14), ♀♀ 0.56–0.73 mm (0.65 ± 0.06 mm, n = 9); PTrL: ♂♂ 0.65–0.74 mm (0.71 ± 0.03 mm, n = 11), ♀♀ 0.57–0.66 mm (0.63 ± 0.03 mm, n = 7); MsTiL: ♂♂ 0.83–1.10 mm (0.97 ± 0.08 mm, n = 14), ♀♀ 0.78–1.01 mm (0.87 ± 0.07 mm, n = 9); MsTrL: ♂♂ 1.06–1.30 mm (1.16 ± 0.06 mm, n = 11), ♀♀ 0.91–1.13 mm (1.05 ± 0.07 mm, n = 9); MtTiL: ♂♂ 0.69–0.91 mm (0.81 ± 0.05 mm, n = 14), ♀♀ 0.66–0.86 mm (0.76 ± 0.06 mm, n = 9); MtTrL: ♂♂ 1.48–1.87 mm (1.67 ± 0.11 mm, n = 10), ♀♀ 1.33–1.69 mm (1.51 ± 0.11 mm, n = 9); PygL: ♂♂ 1.42–1.85 mm (1.58 ± 0.12 mm, n = 13), ♀♀ 1.17–1.56 mm (1.37 ± 0.12 mm, n = 9); TVtL: ♂♂ 0.54–0.87 mm (0.68 ± 0.10 mm, n = 13), ♀♀ 0.44–0.79 mm (0.63 ± 0.11 mm, n = 9); LPrL: 0.29–0.35 mm (0.31 ± 0.02 mm, n = 14); RPrL: 0.22–0.26 mm (0.24 ± 0.01 mm, n = 14); St8L: ♂♂ 0.57–0.63 mm (n = 2); St8W: ♂♂ 0.38–0.40 mm (n = 2).

Habitus illustrated in Fig. 1. Body slender, widest at the end of anterior third of elytra. Integument black. Head and pronotum covered with yellowish pubescence; pubescence on elytra yellowish in proximal half, gradually darkened towards apices, sometimes with reddish or violet metallic sheen; venter covered with yellowish pubescence, darkened along posterior margins of ventrites 3–5.

Head moderately convex dorsally, wider than long, widest before middle, HW/HL ratio: ♂♂ 1.10–1.23 (1.17 ± 0.04, n = 14), ♀♀ 1.13–1.23 (1.17 ± 0.03, n = 9). Dorsal surface with microreticulation and small round punctures bearing short setae; ventral surface with transverse microreticulation and sparse, small punctures bearing short setae; small medial triangular part before gula without punctures. Occipital margin rounded in dorsal aspect, straight or slightly concave if seen from behind. Eyes oval, finely faceted with short interfacetal setae. Anterior margin of clypeus straight. Labrum transverse, approximately two times as wide as long, anterior margin straight; surface with microreticulation and small, round punctures bearing short setae. Antennae rather long, slightly serrate (Fig. 5A, B); antennomeres I–IV subequal in length, slightly shorter and slenderer than following ones; antennomeres V–X in males 1.40–1.60×, in females 1.20–1.30× as long as wide; antennomere XI oval, ~2.30× as long as wide. Mandibles symmetrical, bidentate, lateral portions microreticulated with short setae, outer distal portion with group of seven long sensilla; mola well developed, minutely dentate; prostheca well developed, setose. Galea gradually expanded toward apex, covered with apically expanded sensilla; lacinia setose medio-apically, reaching half of length of galea. Maxillary palpomere II distinctly expanded with long setae on ventral side in males (Fig. 4A); not expanded, without long setae in females (Fig. 4B); maxillary palpomere III short, ~1.50× as long as wide; terminal maxillary palpomere broadly securiform, inner angle situated around middle, TPalL/TPalW ratio: ♂♂ 1.95–2.20 (2.09 ± 0.09, n = 14), ♀♀ 1.95–2.20 (2.10 ± 0.08, n = 9). Terminal labial palpomere fusiform, bearing sparse long sensilla on whole surface, and group of short sensilla at apex (Fig. 5C).

Pronotum moderately convex, approximately as long as wide, widest just behind middle, PW/PL ratio: ♂♂ 1.00–1.09 (1.05 ± 0.03, n = 14), ♀♀ 1.02–1.07 (1.06 ± 0.02, n = 9). Surface finely microreticulate, with small, rasp-like punctures bearing flat seta. Anterior margin rounded, slightly produced mesally, anterior angles broadly rounded; lateral carinae emarginated in lateral aspect; posterior margin forming short mesal lobe, emarginated before posterior angles; posterior angles rectangular in lateral aspect. Posterior marginal bead interrupted before posterior angles. Hypomeron triangular with round concavity posteriorly. Prosternum in front of procoxae narrow, expanded laterally; prosternal process incomplete, narrow, slightly constricted in the middle. Scutellar shield small, triangular, covered with small, round punctures bearing short setae. Mesoventral process ~0.50× as wide as mesofemur, parallel-sided, truncate at apex. Metaventrite strongly convex in the middle; surface weakly microreticulated with small, transversally confluent, rasp-like punctures; posterior margin in the produced mesally; discrimen rather indistinct. Metanepisternum trapezoidal, narrowed posteriorly, dorsal margin emarginated, ventral margin straight.

Elytra long, narrow, widest in anterior 1/3, EL/EW ratio: ♂♂ 2.02–2.26 (2.12 ± 0.08, n = 14), ♀♀ 1.88–2.07 (2.01 ± 0.05, n = 9). Surface with weak transverse microreticulation and rasp-like punctures bearing flat setae. Lateral margins regularly rounded, apices separately rounded.

Protibiae in males expanded basally, bearing fringe of long setae in basal 1/3; PTiL/PTrL ratio: ♂♂ 1.02–1.17 (1.08 ± 0.05, n = 11), ♀♀ 0.95–1.12 (1.03 ± 0.05, n = 7). Protarsomere I as long as two following tarsomeres combined; protarsomere IV simple, parallel-sided, shallowly emarginate at apex. Claws on protarsi with three, on meso and metatarsi with four denticles. Mesotibiae slightly bent inwards; mesotarsus longer than tibia, MsTiL/MsTrL ratio: ♂♂ 0.78–0.89 (0.82 ± 0.03, n = 11), ♀♀ 0.79–0.90 (0.83 ± 0.03, n = 9). Metacoxae large, anterior margin straight, posterior margin broadly rounded. Metatibiae bearing short subapical ridge and 3–4 lateral ridges parallel with apical margin of tibia, reaching 1/3 of tibial width. Metatibial spurs black, inner one ~1.30× as long as outer one. Metatarsomere I bearing 4–5 short ridges, metatarsomere II bearing 2–3 ridges, metatarsomeres III and IV without ridges. Metatarsus ~2.00× as long as metatibia, MtTrL/MtTiL ratio: ♂♂ 1.97–2.18 (2.07 ± 0.06, n = 10), ♀♀ 1.89–2.11 (1.98 ± 0.06, n = 9).

Pygidium long, slender, narrowly truncate at apex, PygL/TVtL ratio: ♂♂ 2.00–3.04 (2.36 ± 0.26, n = 13), ♀♀ 1.76–3.14 (2.22 ± 0.37, n = 9). Apical margin of terminal abdominal ventrite arcuate.

Male genitalia: sternite VIII with long setae in apical part, apical margin produced and weakly bilobed mesally (Fig. 5I), St8L/St8W ratio: ♂♂ 1.49–1.59 (n = 2). Sternite IX long, slender, arrow-shaped, with medial longitudinal keel at apex. Parameres (Fig. 5D–G) rather short, EL/LPrL ratio: 7.87–9.17 (8.48 ± 0.40, n = 14); EL/RPrL ratio: 10.07–11.89 (11.10 ± 0.50, n = 14); LPrL/RPrL ratio: 1.24–1.37 (1.31 ± 0.04, n = 14). Left paramere with short basal part, LPrL/BLPr ratio: 1.88–2.12 (1.98 ± 0.08, n = 14); dorsal branch expanded and obliquely truncate apically; ventral branch slender, slightly bent medially, pointed at apex. Right paramere rather short, ventral branch distinctly shorter than dorsal one, dorsally bent; dorsal branch expanded and rounded apically. Median lobe long, slender, apical part slightly expanded (Fig. 5H). Phallobase with short tubular process (approximately 1/6 of total length) and long, slender furca.

Female genitalia: sternite VIII with apical protuberance and long setae alongside apical and lateral margins, spiculum ventrale short, broadly clavate (Fig. 5J); St8L/St8W ratio: ♀ 1.36 (n = 1).

##### Sexual dimorphism.

Females are usually more robust; with shorter antennae. Maxillary palpomere II is not expanded in females and without long setae on ventral side. Terminal maxillary palpomere is shorter in females, with angles more rounded. Protibiae are not expanded in females, without long setae in basal portion.

##### Distribution.

Croatia, Cyprus, France, Greece, Iran, Israel, Jordan, Macedonia, Montenegro, Romania, Spain, Syria, Turkey, Turkmenistan, Ukraine. *Mordellistenahirtipes* is reported here for the first time from Croatia, Montenegro, and Spain. Csiki (1915) mentioned also “Österreich” (Austria); however, this information is probably based on a misidentification.

##### Biology.

Adults were found on the flowers of *Daucus* sp. (Apiaceae) and *Helichrysum* sp. (Asteraceae) on dry grasslands and in urban environment.

##### Remarks.

Franciscolo (1949) described *M.aegea* based on three specimens from Kos island (Greece). Batten (1977) mentioned that this species does not belong to the *micans* group because the antennomere IV and V are equal in length. Examination of holotype revealed that this specimen belongs to *M.hirtipes*. We consider this taxon as a junior synonym of *M.hirtipes*.

Czető (1990) described *M.podlussanyi* based on a single male specimen from Crete. He mentioned in the original description that the maxillary palpomere II is not dilated. Examination of the holotype actually revealed, that the palpomere is expanded, and any other differences which could separate this taxon from *M.hirtipes* were found. This interpretation is also supported by the results of PCA analysis (Fig. 10A). We propose *M.podlussanyi* as a junior synonym of *M.hirtipes*.

In HNHM collections, there are three specimens of *M.hirtipes*, labelled by Reitter as holotype and paratypes. However, these specimens are not mentioned in original description and labels were probably added subsequently, after the description. These specimens are not parts of the type series.

#### Mordellistena (s. str.) pseudohirtipes

Taxon classificationAnimaliaColeopteraMordellidae

Ermisch, 1965

Figs 2 , 6A–J

Mordellistena pseudohirtipes Ermisch, 1965: 268 (original description); Batten 1976: 168 (distribution); Batten 1977: 171–173 (distribution, figures, key); Plaza 1983: 574 (distribution, biology); Czető 1990: 28–29 (description, figure); Franciscolo 1995: 12 (distribution); Horák 2008: 100 (distribution); Odnosum 2010: 153, 194–195 (key, description, distribution); Ruzzier 2013: 109 (distribution).
Mordellistena fageli Ermisch, 1969: 112 syn. nov. (original description); Batten 1976: 168 (distribution); Plaza 1983: 575–576 (distribution, biology); Horák 1983: 13 (remarks); 2008: 97 (distribution).
Mordellistena pseudohirtipes krotosensis Czető, 1990: 28 syn. nov. (original description); Horák 2008: 100 (distribution).

##### Type locality.

Nessebar env., Bulgaria.

##### Type material examined.

*M.pseudohirtipespseudohirtipes*: HOLOTYPE: 1 ♂, SNSD: “♂ / Genitalpräparat / Bulgaria Umg. Nessebar Juli 1961 leg. BECH / Holotypus [red label] / MORDELLISTENA pseudohirtipes Erm. K Ermisch det. 19 / Coll. ERMISCH Leipzig Ankauf 1970 / Staatl. Museum für Tierkunde Dresden”; PARATYPE: 1 ♀, SNSD: “♀ / Bulgaria Umg. Nessebar Juli 1961 leg. BECH / Allotypus [red label] / MORDELLISTENA pseudohirtipes Erm. K Ermisch det. 19 / Coll. ERMISCH Leipzig Ankauf 1970 / Staatl. Museum für Tierkunde Dresden”. *M.pseudohirtipeskrotosensis*: HOLOTYPE: 1 ♂, HNHM: “[transparent plastic board with dissected genitalia] / ♂ / Krotos KRÉTA / 1981. V. 12. leg. Podlussány / Holotypus Mordellistenapseudohirtipes Ermisch, 1965 ssp. krotosensis, Czető 1988 [red label] / Mordellistena (s. str.) pseudohirtipes Ermisch, 1965 D. Selnekovič det. 2017”. *M.fageli*: HOLOTYPE: 1 ♂, SNSD: “♂ / Genitalpräparat / Portugal: Algrave Caldas de Monchique V–1960 G. Fagel / R. I. Sc. N. B. I. G. 22.145 / Holotypus [red label] / coll. ERMISCH, Leipzig, Ankauf 1970 / Staatl. Museum für Tierkunde Dresden / HOLOTYPUS Mordellistena (s. str.) fageli Ermisch, 1969 D. Selnekovič labelled 2017 [red label] / Mordellistena (s. str.) pseudohirtipes Ermisch, 1965 D. Selnekovč det. 2017”; PARATYPE: 1 ♀, SNSD: “♀ / Portugal: Algarve Caldas de Monchique V–1960 G. Fagel / R. I. Sc. N. B. I. G. 22.145 / Allotypus [red label] / coll. ERMISCH, Leipzig, Ankauf 1970 / Staatl. Museum für Tierkunde Dresden / ALLOTYPUS (PARATYPUS) Mordellistena (s. str.) fageli Ermisch, 1969 D. Selnekovič labelled 2017 [red label] / Mordellistena (s. str.) pseudohirtipes Ermisch, 1965 D. Selnekovič det. 2017”.

##### Additional material examined.

**Algeria.** 1 ♂, SNSD: “Algérie: Algérois, Kaddous 3–V–1954 G. Fagel / R. I. Sc. N. B. I. G. 19.867 / coll. ERMISCH, Leipzig, Ankauf 1970 / Staatl. Museum für Tierkunde Dresden / “Mordellistena (s. str.) pseudohirtipes Ermisch, 1965 D. Selnekovič det. 2017” [in collection as *M.fageli*]. **Bulgaria.** 1 ♂, SNSD: “♂ / Nessebar, Bulgaria 28. 5. – 10. 6. 1963 Karl Bleyl / MORDELLISTENA pseudohirtipes Erm. K. Ermisch det. 19”; 1 ♂, CSB: “Bulgaria mer. occ. Sandanski (→ Liljanovo) 5. – 10. 1976 Karel Majer lgt. / Mordellistena (s. str.) pseudohirtipes Ermisch, 1965 D. Selnekovič det. 2016”. **France.** 2 ♂♂, SNSD: “♂ / Genitalpräparat / France Basses Alpes St. Michel l’Observat. 24. 7 – 10. 8. 63 Rudkjöb. / MORDELLISTENA pseudohirtipes Erm. K. Ermisch det. 19”; 1 ♂, SNSD: “♂ / Ardêche 10. 7. 65 Banne Balazuc / MORDELLISTENA pseudohirtipes Erm. K. Ermisch det. 19; 1 ♂ SNSD: “♂ / Genitalpräparat / Südfrankreich Camargue, 13. 6. 1952, leg. Freude / Mordellistena Lopezi Ermisch det. K. Ermisch 63” [in collection as *M.lopezi*]; 1 ♂, SNSD: “♂ / Genitalpräparat / Pyrenées or. Umg. Banyuls 30. 5.–10. 6. 53 / Mordellistena Lopezi Ermisch det. K. Ermisch 63” [in collection as *M.lopezi*]; 1 ♂, SNSD: “Banyuls Pyr. or. VI. 53 J. u. B. Bechyne / Museum Frey München” [in collection as *M.lopezi*]; 1 ♂, SNSD: “♂ / Gall. mer. Agay (Var) 18. 7. 58 W. Liebmann / Genitalpräparat” [in collection as *M.lopezi*]; 1 ♂, SNSD: “♂ / Genitalpräparat / Fr. Ardêche Bois de Paiolive 1. 7. 66 Balazuc [hand written] / Paratypus / Staatl. Museum für Tierkunde Dresden / PARATYPUS Mordellistena (s. str.) geronensis Ermisch, 1977, Selnekovič labelled 2017 [red label] / Mordellistena (s. str.) pseudohirtipes Ermisch, 1965 D. Selnekovič det. 2017” [in collection as *M.geronensis*]. **Georgia.** 1 ♂, SNSD: “♂ / Genitalpräparat / SSSR–Gruzie Tbilisi 7.57 R. Dvořák / MORDELLISTENA pseudohirtipes Erm. K. Ermisch det. 19 / Coll. ERMISCH Leipzig Ankauf 1970 / Staatl. Museum für Tierkunde Dresden”; 2 ♂♂, SNSD: “♂ / SSSR–Gruzie Tbilisi 7.57 R. Dvořák / MORDELLISTENA pseudohirtipes Erm. K. Ermisch det. 19”. **Greece.** 1 ♂, SNSD: “♂ / Genitalpräparat / Athos Daphni A. Schatzmayr / MORDELLISTENA pseudohirtipes Erm. K Ermisch det. 19”; 1 ♂, SNSD: “♂ / Genitalpräparat / Ephesus / J. Sahlb. / MORDELLISTENA pseudohirtipes Erm. K. Ermisch det. 19”; 1 ♂, HNHM: “Creta Biró / Amari 4. VII. 06 / Mordellistenapseudohirtipes Erm. det. R. Batten 1980 / Mordellistena (s. str.) pseudohirtipes Ermisch, 1965 D. Selnekovič det. 2017”. **Israel**. 1 ♂, CSB: “Israel Jerusalem 25. III. 2001, ??? leg. / Mordellistena s. str. pseudohirtipes Ermisch, 1965 D. Selnekovič det. 2014”; 1 ♂, HNHM: “Izrael Rehovot 1965. V. 20. Dr. Erdös / coll. Dr. J. Erdös / Mordellistenapseudohirtipes Erm. det. R. Batten 1980 / Mordellistena (s. str.) pseudohirtipes Ermisch, 1965 D. Selnekovič det. 2017”. **Italy.** 1 ♂, SNSD: “♂ / Genitalpräparat / Sicilia Magara d. V. 16. 5. 61 W. Liebmann” [in collection as *M.lopezi*]. **Macedonia.** 1 ♂, SNSD: “♂ / Veles, Mac. 23. – 25. 5. 55. leg. F. Schubert / Genitalpräparat / MORDELLISTENA pseudohirtipes Erm. K Ermisch det. 19”. **Montenegro**. 2 ♂♂, 1 ♀, CSB: “Montenegro S Skadarske jazero lake N, Virpazar village env. D. Selnekovič 21. VI. 2011 / Mordellistenapseudohirtipes Ermisch, 1965 D. Selnekovič det. 2012”. **Spain.** 1 ♂, SNSD: “♂ / Genitalpräparat / Son Españolet 1–VI–1958 R. López / Paratypus [red label] / Mordellistena Lopezi Ermisch det. K. Ermisch / Mordellistena (s. str.) pseudohirtipes Ermisch, 1965 D. Selnekovič det. 2017” [in collection as *M.lopezi*]; 1 ♂, SNSD: “♂ / Genitalpräparat / SEVILLA Hi. m. Marismas, V. 1943 G. Frey, C. Koch / Mordellistena Lopezi Ermisch det. K. Ermisch 63 / Mordellistena (s. str.) pseudohirtipes Ermisch, 1965 D. Selnekovič det. 2017” [in collection as *M.lopezi*]; 1 ♂, CSB: “Spain, Málaga, Lagunas de Archidona, 800m 37°06'N, 04°18'40"W, 12.–14. V. 2018 E. Jendek / Mordellistena (s. str.) pseudohirtipes Ermisch, 1965 D. Selnekovič det. 2018”. **Ukraine.** 2 ♂♂, SNSD: “♂ / Genitalpräparat / Umgeb. Jalta Krim, Ende Juli 1965 leg. F. Hieke / MORDELLISTENA pseudohirtipes Erm. K Ermisch det. 19”; 1 ♂, HNHM: “Krim Jaila 17. VI. 1956 leg. L. Horváth / Mordellistenapseudohirtipes Erm. det. R. Batten 1980 / Mordellistena (s. str.) pseudohirtipes Ermisch, 1965 D. Selnekovič det. 2017”.

##### Differential diagnosis.

From *M.purpurascens* it differs in shorter parameres (EL/LPrL ratio: *M.pseudohirtipes*: 4.65–7.17 (5.89 ± 0.71, n = 25), *M.purpurascens*: 4.42–5.84 (4.98 ± 0.35, n = 19); EL/RPrL ratio: *M.pseudohirtipes*: 5.91–8.63 (7.42 ± 0.72, n = 25), *M.purpurascens*: 5.57–6.94 (6.19 ± 0.41 n = 19)). Basal part of the left paramere (Fig. 6E–H) is shorter than in *M.purpurascens* (Fig. 7G–J). Body usually smaller (TL: *M.pseudohirtipes*: ♂♂ 2.47–4.05 mm (3.20 ± 0.43 mm, n = 25) ♀♀ 3.26–4.47 mm (3.68 ± 0.56 mm, n = 3), *M.purpurascens*: ♂♂ 3.10–4.42 mm (3.75 ± 0.35 mm, n = 19), ♀♀ 3.31–4.42 mm (3.82 ± 0.36 mm, n = 14)). Differences between *M.hirtipes* are mentioned above.

##### Redescription.

Measurements: TL: ♂♂ 2.47–4.05 mm (3.20 ± 0.43 mm, n = 25) ♀♀ 3.26–4.47 mm (3.68 ± 0.56 mm, n = 3); HL: ♂♂ 0.64–0.93 mm (0.75 ± 0.09 mm, n = 25), ♀♀ 0.77–1.04 mm (0.87 ± 0.12 mm, n = 3); HW: ♂♂ 0.70–1.06 mm (0.87 ± 0.11 mm, n = 29), ♀♀ 0.87–1.20 mm (0.99 ± 0.15 mm, n = 3); PL: ♂♂ 0.83–1.29 mm (1.05 ± 0.14 mm, n = 25), ♀♀ 1.06–1.42 mm (1.19 ± 0.16 mm, n = 3); PW: ♂♂ 0.83–1.39 mm (1.07 ± 0.17 mm, n = 29), ♀♀ 1.12–1.67 mm (1.31 ± 0.25 mm, n = 3); EL: ♂♂ 1.88–3.02 mm (2.37 ± 0.31 mm, n = 25), ♀♀ 2.44–3.35 mm (2.75 ± 0.42 mm, n = 3); EW: ♂♂ 0.81–1.46 mm (1.09 ± 0.18 mm, n = 25), ♀♀ 1.17–1.69 mm (1.35 ± 0.24 mm, n = 6); PTiL: ♂♂ 0.57–0.91 mm (0.70 ± 0.10 mm, n = 25), ♀♀ 0.65–0.90 mm (0.74 ± 0.11 mm, n = 6); PTrL: ♂♂ 0.52–0.84 mm (0.63 ± 0.08 mm, n = 24), ♀♀ 0.60–0.80 mm (0.68 ± 0.09, n = 3); MsTiL: 0.71–1.17 mm (0.87 ± 0.12 mm, n = 25), ♀♀ 0.86–1.25 mm (0.99 ± 0.18 mm, n = 3); MsTrL: ♂♂ 0.83–1.34 mm (1.02 ± 0.15 mm, n = 13), ♀♀ 1.04–1.35 mm (n = 2); MtTiL: ♂♂ 0.60–0.92 mm (0.74 ± 0.09 mm, n = 25), ♀♀ 0.74–1.04 mm (0.84 ± 0.14 mm, n = 3); MtTrL: ♂♂ 1.27–1.98 mm (1.59 ± 0.20 mm, n = 17), ♀♀ 1.51–2.05 mm (n = 2); PygL: ♂♂ 1.29–1.96 mm (1.54 ± 0.18 mm, n = 29), ♀♀ 1.39–1.75 mm (1.57 ± 0.14 mm, n = 3); TVtL: ♂♂ 0.56–1.25 mm (0.73 ± 0.13 mm, n = 25), ♀♀ 0.50–0.71 mm (0.64 ± 0.10 mm, n = 3); LPrL: 0.33–0.46 mm (0.40 ± 0.03 mm, n = 25); RPrL: 0.26–0.37 mm (0.32 ± 0.03 mm, n = 25); St8L: ♂ 0.47 mm (n = 1); St8W: ♂ 0.31 mm (n = 1).

Habitus given in Fig. 2. Body strongly elongate, slender, widest just behind humeri. Integument black, mouthparts sometimes paler. Pubescence on head pale yellowish, on pronotum yellowish to dark grey, on elytra yellowish in anterior 1/2, darkened towards apices, or completely dark grey, sometimes with reddish or purplish metallic sheen, on pygidium dark grey, on venter yellowish, darkened along posterior margins of abdominal ventrites.

Head moderately convex dorsally, wider than long, widest just before middle, HW/HL ratio: ♂♂ 1.08–1.23 (1.15 ± 0.03, n = 25), ♀♀ 1.11–1.16 (1.13 ± 0.02, n = 3). Dorsal surface weakly microreticulated with small, round punctures bearing short setae. Occipital margin rounded in dorsal aspect, straight, or slightly concave seen from behind. Eyes oval, completely reaching occiput, not expanded onto ventral surface, finely faceted, with short interfacetal setae. Anterior margin of clypeus straight. Labrum transverse, anterior margin straight or very slightly emarginate; surface microreticulation with small, round punctures and setae. Antennae slightly serrate (Fig. 6A, B); antennomeres I–IV subequal in length; antennomeres V–X longer and wider, in males ~1.30×, in females ~1.20× as long as wide; terminal antennomere elongate oval, ~1.90× as long as wide. Galea gradually expanded toward apex, covered with apically expanded sensilla. Maxillary palpi (Fig. 6C–D) black; palpomere I very short; palpomere II distinctly expanded, with long setae on ventral side in males, not expanded, rather long and narrow in females; palpomere III short, ~1.50× as long as wide; terminal palpomere in males broadly securiform, with inner angle situated around the middle, in females slenderer, with inner angle situated in apical 1/3; TPalL/TPalW ratio: ♂♂ 1.80–2.30 (2.07 ± 0.11, n = 25), ♀♀ 2.05–2.31 (2.21 ± 0.12, n = 3).

Pronotum moderately convex, approximately as long as wide, PW/PL ratio: ♂♂ 0.97–1.12 (1.02 ± 0.03, n = 25), ♀♀ 1.04–1.18 (1.10 ± 0.06, n = 3). Surface finely transversally microreticulate, covered with rasp-like punctures, distance between punctures 2.00–4.00 times as long as the diameter, each puncture bears flat, pointed seta. Anterior margin rounded, slightly produced mesally, anterior angles broadly rounded; lateral carinae emarginated in lateral aspect; posterior margin forming short mesal lobe, emarginated before posterior angles; posterior angles in lateral aspect rectangular, acute. Posterior marginal bead interrupted before posterior angles. Scutellar shield small, triangular, with small, rasp-like punctures bearing setae. Metanepisternum trapezoidal, narrowed posteriorly, ventral margin straight, dorsal margin emarginate.

Elytra long and narrow, moderately convex, widest at the end of anterior 1/4, EL/EW ratio: ♂♂ 2.02–2.40 (2.18 ± 0.10, n = 25), ♀♀ 1.99–2.08 (2.04 ± 0.04, n = 3). Surface with weak transverse microreticulation and rasp-like punctures, these are larger and more densely arranged than those on pronotum, each puncture bears flat seta. Lateral margins rather strongly convergent, regularly rounded; apices separately rounded.

Profemora slender, in males somewhat stouter than in females. Protibiae straight, in males distinctly expanded in basal half, here with fringe of long, thick setae; PTiL/PTrL ratio: ♂♂ 1.02–1.24 (1.11 ± 0.05, n = 25), ♀♀ 1.02–1.13 (1.09 ± 0.05, n = 3). Protarsomere I in females as long as two following tarsomeres combined, in males slightly longer; protarsomere IV simple, slightly shorter than previous one, shallowly emarginate at apex; terminal protarsomere slightly shorter than previous two tarsomeres combined. Claws on protarsi with three denticles, on meso- and metatarsi with four denticles. Mesotibiae slightly bent medially; mesotarsus longer than tibia, MsTiL/MsTrL ratio: ♂♂ 0.79–0.90 (0.83 ± 0.03, n = 13), ♀♀ 0.85–0.92 (n = 2). Metacoxae large, anterior margin slightly emarginated, posterior margin broadly rounded. Metatibiae bearing short subapical ridge and 3–4 lateral ridges parallel to apical margin of tibia, reaching 1/3 of tibial width. Metatibial spurs black, long, inner one ~1.30× as long as outer one. Metatarsomere I bearing 3–5 short ridges, metatarsomere II bearing 2–3 ridges, metatarsomere III without ridges. Metatarsus ~2.00× as long as metatibia, MtTrL/MtTiL ratio: ♂♂ 2.02–2.32 (2.18 ± 0.07, n = 17), ♀♀ 1.98–2.00 (n = 2).

Pygidium long and slender, PygL/TVtL ratio: ♂♂ 1.82–2.47 (2.19 ± 0.15, n = 23), ♀♀ 1.88–2.79 (2.22 ± 0.32, n = 6). Apical margin of terminal ventrite arcuate.

Male genitalia: sternite VIII rather short, setae present in apical 1/3, apical protuberance short, slightly bilobed at apex (Fig. 6J); St8L/St8W ratio: ♂♂ 1.50–1.53 (n = 2). Sternite IX long, slender, arrow-shaped, with medial longitudinal keel in apical part. Parameres (Fig. 6E–H) rather long, EL/LPrL ratio: 4.65–7.17 (5.89 ± 0.71, n = 25), EL/RPrL ratio: 5.91–8.63 (7.42 ± 0.72, n = 25), LPrL/RPrL ratio: 1.12–1.39 (1.26 ± 0.06, n = 25). Left paramere: dorsal branch expanded apically, obliquely truncate at apex; ventral branch narrow, slightly bent medially, pointed at apex, LPrL/BLPr ratio: 1.73–2.24 (1.95 ± 0.11, n = 25). Right paramere: dorsal branch rather narrow, slightly expanded, rounded at apex; ventral branch as long as or slightly shorter than dorsal branch, bent dorsally in apical part, pointed at apex. Median lobe long, slender, slightly expanded in apical part (Fig. 6I). Phallobase with short tubular process (approximately 1/6 of total length) and long, slender furca.

##### Sexual dimorphism.

Females are more robust than males, their protibiae are not expanded in basal 1/3 and without fringe of long setae. Maxillary palpomere II is not expanded in females and without long setae on ventral side. Terminal maxillary palpomere is slenderer in females and its inner angle is situated more distally than in males. Antennae are somewhat shorter in females.

##### Distribution.

Algeria, Azerbaijan, Bulgaria, Croatia, Cyprus, France, Georgia, Greece, Israel, Italy, Macedonia, Montenegro, Morocco, Portugal, Spain, Turkey, Ukraine. *Mordellistenapseudohirtipes* is reported here for the first time from Israel and Montenegro.

##### Biology.

Adults were collected on the flowers of Apiaceae plants on dry grasslands. Plaza (1983) mentioned that *M.pseudohirtipes* was collected in Spain on following plant species: *Thapsiavillosa*, *Daucuscarota* (both Apiaceae) and *Rutamontana* (Rutaceae).

##### Remarks.

*Mordellistenafageli* was placed in the *pentas*–group in the original description, based on the dark pubescence and three ridges on the metatarsomere II. In fact, as Horák (1983) already mentioned, this species belongs to *M.hirtipes* complex, based on the strongly convex body and expanded maxillary palpomere II. Examination of type material did not reveal any characters which could separate this taxon from *M.pseudohirtipes*. In the plot from PCA analysis (Fig. 10A), *M.fageli* is placed just next to the cluster of *M.pseudohirtipes*, in the same plane along the PC 1 axis. We consider these taxa as conspecific and propose *M.fageli* as a junior synonym of *M.pseudohirtipes*.

Czető (1990) described *M.pseudohirtipeskrotosensis* based on two male specimens from Crete island. Characters such as length of the body, length of pygidium and colouration of pubescence, that he used for differentiation of the subspecies, are subjects of individual variability. Results of PCA (Fig. 10A) show, that holotype of *M.pseudohirtipeskrotosensis* is placed within the cluster of the nominotypical subspecies. After examination of holotype we consider this subspecies as a junior synonym of *M.pseudohirtipespseudohirtipes*.

In Ermisch’s collection, there is a series of specimens named *Mordellistenalopezi*. Such species has not been described, and in fact, all the specimens belong to *M.pseudohirtipes*, except the one labelled as “Type”, which belongs to *M.purpurascens*.

#### Mordellistena (s. str.) purpurascens

Taxon classificationAnimaliaColeopteraMordellidae

Costa, 1854

Figs 3 , 7A–K

Mordellistena purpurascens Costa, 1854: 17 + Plate XXI (original description, figure); Gemminger and Harold 1870: 2113 (catalogue, as syn. of Mordellistenapumila (Gyllenhal, 1810)); Emery 1876: 95 (as syn. of Mordellistenamicans (Germar, 1817)); Baudi di Selve 1877: 827 (as syn. of M.micans); Heyden et al. 1883: 142 (catalogue, as syn. of M.micans); Schilsky 1898: 77 (as syn. of M.micans); Heyden et al. 1906: 456 (catalogue, as syn. of M.micans); Csiki 1915: 37 (catalogue, as syn. of M.micans); Ermisch 1977: 169 (misidentification); Batten 1977 (misidentification); Kaszab 1979: 72, 74 (misidentification); Franciscolo 1991: 172–173 (remarks); Odnosum 2003: 36–46 (misidentification); Odnosum 2005: 100–108 (misidentification); Horák 2008: 100 (misidentification); Odnosum 2010: 199 (misidentification); Ruzzier 2013: 110 (misidentification).
Mordellistena geronensis Ermisch, 1977: 169 syn. nov. (original description in the key); Kaszab 1979: 72–73 (figures, key); Franciscolo 1991: 171–172 (key, figures); Horák 2008: 98 (distribution).
Mordellistena istrica Ermisch, 1977: 169 syn. nov. (original description in the key); Kaszab 1979: 73 (key); Horák 2008: 98 (distribution).

##### Type locality.

Naples, Italy.

##### Type material examined.

*M.purpurascens*: LECTOTYPE, here designated, glued, genitalia in separate microvial, right metatarsus missing: 1 ♂, MZFN: “Mordellistenapurpurascens n. Napoli [hand written by Costa] / LECTOTYPE Mordellistenapurpurascens Costa, 1854 D. Selnekovič des. 2019” [red label]. *M.geronensis*: HOLOTYPE: 1 ♂, SNSD: “♂ / Genitalpräparat / [card with dissected genitalia, right antenna and right protarsus] / Spanien, Prov. Gerona, Tossa de mar, A. Kampf. VII–VIII 35 / Holotypus [red label] / Holotypus Mordellistenageronensis Ermisch / Staatl. Museum für Tierkunde Dresden”; PARATYPES: 1 ♀, SNSD: “Spanien, Prov. Gerona, Tossa de mar, A. Kampf, V–VI 35 / sp.? grupe micans, det. Ermisch 1940 / Paratypus / PARATYPUS Mordellistena (s. str.) geronensis Ermisch, 1977, Selnekovič labelled 2017 [red label] / Mordellistena (s. str.) purpurascens Costa, 1854, D. Selnekovič det. 2019”; 2 ♂♂, 2 ♀♀, SNSD: “♂ [or] ♀ / Ardêche, 10. 7. 65, Banne, J. Balazuc / Paratypus / PARATYPUS Mordellistena (s. str.) geronensis Ermisch, 1977, Selnekovič labelled 2017 [red label] / Mordellistena (s. str.) purpurascens Costa, 1854, D. Selnekovič det. 2017”; 1 ♂, SNSD: “♂ / Ardêche, Sammzon, 8. 7. 65, Balazuc / Paratypus / PARATYPUS Mordellistena (s. str.) geronensis Ermisch, 1977, Selnekovič labelled 2017 [red label] / Mordellistena (s. str.) purpurascens Costa, 1854, D. Selnekovič det. 2017”. *M.istrica*: HOLOTYPE: 1 ♂, SNSD: “♂ / [card with dissected genitalia] / Pola, Istr. F. Lang / [blank red circular label] / MORDELLISTENA istrian [illegible handwriting] det. Ermisch 1952 / Type [red label] / Holotypus Mordellistenaistrica Ermisch / Mordellistena (Mordellistena) geronensis Ermisch det. P. Leblanc 2007 / Mordellistena (s. str.) purpurascens Costa, 1854 D. Selnekovič det. 2019”; PARATYPES: 1 ♀, SNSD: “Plomin, Warmehang 14. 6. 1965 / Istrien K. Wellschmeld / Paratypus / PARATYPUS Mordellistena (s. str.) istrica Ermisch, 1977, Selnekovič labelled 2017” [red label]; 1 ♂, SNSD: “♂ / Corsica / Paratypus / PARATYPUS Mordellistena (s. str.) istrica Ermisch, 1977, Selnekovič labelled 2017 [red label] / Mordellistena (Mordellistena) geronensis Ermisch det. P. Leblanc 2007 / Mordellistena (s. str.) purpurascens Costa, 1854 D. Selnekovič det. 2019”; 1 ♂, 1 ♀, SNSD: “12. 7. 14 Gallia m. Agay Rapp / Paratypus / PARATYPUS Mordellistena (s. str.) istrica Ermisch, 1977, Selnekovič labelled 2017” [red label].

##### Additional material examined.

**Greece**: 1 ♂, 2 ♀♀, CSB: “Greece N, Corfu – Kavos, 39°24'16"N, 20°05'53"E, F. Repta leg., 28. VIII. 2011 / Mordellistenapurpurascens Costa, 1854, D. Selnekovič det. 2019”. **Italy**: 1 ♂, CSB: “IT–Sicilia, Madonia, Termini, Sciara, M San Calogero, ex. l, 2.–3. 6. 2011, M. Šárovec, 3. 8 / Mordellistenapurpurascens Costa, 1854, D. Selnekovič det. 2019”; 1 ♂, SNSD: “♂ / Gavoi Sard. 750m 21.–26. 8. 55 J. Kless 78 / Mordellistena Lopezi Ermisch det. K. Ermisch / Mordellistena (s. str.) purpurascens Costa, 1854 D. Selnekovič det. 2019” [in collection as *M.lopezi*]; 1 ♂, SNSD: “♂ / Genitalpräparat / ITALIA mer. Capaccio Hüdepohl VI. 64 / Mordellistena (s. str.) purpurascens Costa, 1854 D. Selnekovič det. 2019” [in collection as *M.lopezi*]. **Montenegro**: 4 ♂♂, 4 ♀♀ CSB: “Montenegro SE, 42°06'N, 19°06'E, Bar–centrum, on Daucus sp., D. Selnekovič 19. VI. 2011 / Mordellistenapurpurascens Costa, 1854, D. Selnekovič det. 2019”; 1 ♀ CSB: “Montenegro SE, BAR env., 42°07'56"N, 19°07'33"E, 22. VI. 2011 / Mordellistena (s. str.) purpurascens Costa, 1854, D. Selnekovič det. 2019”; 1 ♂ HNHM: “Dalmatia Horváth / Zelenika 906. VIII. / Mordellistenapseudohirtipes Erm. det. R. Batten 1979 / Mordellistena (s. str.) purpurascens, Costa, 1854 D. Selnekovič det. 2019”. **Morocco**: 1 ♂, CSB: Morocco Moyen Atl, Khenifra 15km E M. Šárovec 11. VII. 2007 / Mordellistenapurpurascens Costa, 1854 D. Selnekovič det. 2019”; 4 ♂♂, 3 ♀♀, CSB: “Morocco Moyen Atl, Khenifra 10km I M. Šárovec 30. V. 2007 / Mordellistenapurpurascens Costa, 1854 D. Selnekovič det. 2019”. **Spain**: 1 ♂, SNSD: “♂ / Genitalpräparat / Son Españolet 1–VI–1958 R. López / Typus [red label] / Mordellistena Lopezi Ermisch det. K. Ermisch / Mordellistena (s. str.) purpurascens Costa, 1854 D. Selnekovič det. 2019” [in collection as *M.lopezi*].

##### Differential diagnosis.

*M.purpurascens* closely resembles *M.hirtipes* and *M.pseudohirtipes*. The differences are described under these species.

##### Redescription.

Measurements: TL: ♂♂ 3.10–4.42 mm (3.75 ± 0.35 mm, n = 19), ♀♀ 3.31–4.42 mm (3.82 ± 0.36 mm, n = 14); HL: ♂♂ 0.77–0.97 mm (0.85 ± 0.06 mm, n = 19), ♀♀ 0.78–0.96 mm (0.86 ± 0.06 mm, n = 14); HW: ♂♂ 0.91–1.17 mm (1.01 ± 0.07 mm, n = 19), ♀♀ 0.84–1.12 mm (0.98 ± 0.09 mm, n = 14); PL: ♂♂ 1.06–1.44 mm (1.23 ± 0.10 mm, n = 19), ♀♀ 1.04–1.44 mm (1.25 ± 0.11 mm, n = 14); PW: 1.10–1.56 mm (1.30 ± 0.13 mm, n = 19), ♀♀ 1.13–1.58 mm (1.35 ± 0.14 mm, n = 13); EL: ♂♂ 2.44–3.35 mm (2.81 ± 0.27 mm, n = 19), ♀♀ 2.50–3.38 mm (2.88 ± 0.27 mm, n = 14); EW: ♂♂ 1.15–1.59 mm (1.35 ± 0.13 mm, n = 19), ♀♀ 1.19–1.66 mm (1.43 ± 0.15 mm, n = 14); ATiL: ♂♂ 0.71–0.93 mm (0.81 ± 0.07 mm, n = 19), ♀♀ 0.65–0.91 mm (0.75 ± 0.08 mm, n = 14); ATrL: ♂♂ 0.64–0.84 mm (0.72 ± 0.07 mm, n = 15), ♀♀ 0.62–0.80 mm (0.69 ± 0.06 mm, n = 13); ITiL: ♂♂ 0.91–1.23 mm (1.03 ± 0.10 mm, n = 19), ♀♀ 0.86–1.23 mm (1.01 ± 0.12 mm, n = 14); ITrL: ♂♂ 1.12–1.64 mm (1.27 ± 0.15 mm, n = 9), ♀♀ 1.02–1.34 mm (1.16 ± 0.10 mm, n = 14); PTiL: 0.78–1.08 mm (0.88 ± 0.08 mm, n = 19), ♀♀ 0.75–1.05 mm (0.88 ± 0.08 mm, n = 14); PTrL: 1.64–2.18 mm (1.87 ± 0.19 mm, n = 9), ♀♀ 1.48–2.16 mm (1.77 ± 0.21 mm, n = 11); PygL: ♂♂ 1.50–2.12 mm (1.86 ± 0.18 mm, n = 19), ♀♀ 1.35–1.98 mm (1.67 ± 0.20 mm, n = 14); TVtL: ♂♂ 0.58–0.87 mm (0.77 ± 0.10 mm, n = 18), ♀♀ 0.60–0.92 mm (0.77 ± 0.08 mm, n = 14); RPrL: 0.52–0.64 mm (0.56 ± 0.03 mm, n = 19); LPrL: 0.41–0.51 mm (0.45 ± 0.03 mm, n = 19); St8L: ♂♂ 0.65–0.80 mm (0.70 ± 0.07 mm, n = 3); St8W: ♂♂ 0.49–0.52 mm (0.50 ± 0.01 mm, n = 3).

Habitus illustrated in Fig. 3. Body strongly elongate, slender, widest behind anterior 1/4 of elytra. Integument black, anterior margin of clypeus and mandibles somewhat paler. Pubescence on head and thorax yellowish; on elytra yellowish in anterior half, gradually darkened apically; on venter yellowish, darkened along posterior margins of ventrites 3 and 4 and completely dark grey on terminal ventrite and pygidium.

Head convex dorsally, wider than long, widest about middle, HW/HL ratio: ♂♂ 1.11–1.23 (1.19 ± 0.03, n = 19), ♀♀ 1.06–1.19 (1.14 ± 0.03, n = 14). Dorsal surface weakly microreticulated, with small, round punctures, each bearing short seta. Ventral surface with weak transverse microreticulation and sparsely arranged, round punctures, each bearing short seta. Occipital margin rounded in dorsal aspect, straight if seen from behind. Eyes oval, completely reaching occiput, not expanded onto ventral surface, finely faceted, with short interfacetal setae. Anterior margin of clypeus straight. Labrum transverse, LabW/LabL: ♂♂ 2.04–2.27 (2.15 ± 0.10, n = 5), ♀♀ 1.88–2.38 (2.21 ± 0.20, n = 5), anterior margin straight or very shallowly emarginate mesally; surface covered with small, round punctures, each bearing seta. Antennae slightly serrate, expanded from antennomere V (Fig. 7A, B); antennomeres I and II short, subequal in length and width; antennomere III equal in length and slightly slenderer than previous two; antennomere IV slightly longer and wider than previous one; antennomeres V–X wider than previous four, in males ~1.60×, in females ~1.30× as long as wide; antennomere XI elongate oval, ~2.20× as long as wide. Galea gradually expanded apically, covered with apically expanded sensilla. Maxillary palpi (Fig. 7C–D) black; palpomere I very short; palpomere II in males expanded with long setae on ventral side, in females slenderer, without long setae; palpomere III short, ~1.80× as long as wide, in males with long setae on ventral side; terminal palpomere securiform, in males wider than in females, inner angle situated around middle in males, in terminal 1/3 in females; TPalL/TPalW ratio: ♂♂ 1.72–2.16 (1.96 ± 0.10, n = 17), ♀♀ 2.08–2.34 (2.21 ± 0.09, n = 14).

Pronotum moderately convex, slightly wider than long, PW/PL ratio: ♂♂ 0.97–1.10 (1.05 ± 0.03, n = 19), ♀♀ 1.00–1.15 (1.07 ± 0.04, n = 13). Surface weakly microreticulated with small, rasp-like punctures, distance between punctures 1.50–2.00× as long as puncture diameter, each puncture bearing flat seta. Anterior margin rounded, slightly produced mesally, anterior angles broadly rounded; lateral carinae rounded in dorsal aspect, shallowly but distinctly emarginate in lateral aspect; posterior margin forming short mesal lobe, emarginated laterally before posterior angles; posterior angles rectangular, pointed in lateral aspect. Posterior marginal bead interrupted before posterior angles. Prosternum in front of procoxae narrow, laterally expanded. Scutellar shield small, triangular, with small punctures bearing short setae. Mesoventral process ca. half as wide as mesofemora. Metaventrite large, posterior margin produced mesally between metacoxae; longitudinal discrimen rather indistinct. Metanepisternum trapezoidal, slightly narrowed posteriorly, dorsal margin emarginate, ventral margin straight.

Elytra long, narrow, widest at end of anterior 1/4, EL/EW ratio: ♂♂ 1.97–2.23 (2.08 ± 0.07, n = 19), ♀♀ 1.83–2.15 (2.02 ± 0.07, n = 14). Dorsal surface covered with weak transverse microreticulation and rasp-like punctures, distance between punctures ~1,50× as long as puncture diameter; each puncture bearing flat seta. Lateral margins regularly rounded, apices separately rounded.

Protibiae straight, basal part in males slightly expanded and bearing distinct fringe of long setae; PTiL/PTrL ratio: ♂♂ 0.98–1.24 (1.13 ± 0.06, n = 15), ♀♀ 0.98–1.17 (1.10 ± 0.05, n = 13). Protarsomere I in females as long as two following tarsomeres combined, in males slightly longer; protarsomere IV simple, parallel-sided, very shallowly emarginated at apex. Claws on protarsi rather long, slender, with three denticles, on meso- and metatarsi with four denticles. Mesotibiae slightly bent medially; mesotarsus longer than tibia, MsTiL/MsTrL ratio: ♂♂ 0.75–0.90 (0.84 ± 0.04, n = 9), ♀♀ 0.82–0.95 (0.88 ± 0.04, n = 14). Metacoxae large, anterior margin straight, posterior margin broadly rounded. Metatibiae bearing short subapical ridge and 3–4 lateral ridges parallel to apical tibial margin, reaching 1/3 of tibial width. Metatibial spurs black, inner one ~1.30× as long as outer one. Metatarsomere I bearing in males 5, in females 3–4 short lateral ridges; metatarsomere II bearing 2–3 ridges; metatarsomeres III and IV without ridges. Metatarsus ~2.00× as long as metatibia, MtTrL/MtTiL ratio: ♂♂ 2.00–2.33 (2.13 ± 0.10, n = 10), ♀♀ 1.90–2.18 (2.01 ± 0.07, n = 11).

Pygidium long, slender, PygL/TVtL ratio: ♂♂ 1.88–3.32 (2.43 ± 0.35, n = 18), ♀♀ 1.88–2.64 (2.18 ± 0.17, n = 14). Apical margin of terminal abdominal ventrite arcuate.

Male genitalia: sternite VIII rather short, with long setae in apical part, apical protuberance rather short, slightly bilobed at apex (Fig. 7E); St8L/St8W ratio: ♂♂ 1.26–1.32 (1.28 ± 0.02, n = 4). Sternite IX long, slender, arrow-shaped, with medial longitudinal keel apically. Parameres (Fig. 7G–J) rather long, EL/LPrL ratio: 4.42–5.84 (4.98 ± 0.35, n = 19), EL/RPrL ratio: 5.57–6.94 (6.19 ± 0.41 n = 19); LPrL/RPrL ratio: 1.16–1.30 (1.24 ± 0.03, n = 19). Left paramere with very long basal part, LPrL/BLPr ratio: 1.50–1.91 (1.76 ± 0.09, n = 19); dorsal branch strongly expanded apically, obliquely truncate at apex; ventral branch slender, slightly bent medially. Right paramere rather long with long branches; ventral branch longer than dorsal one, pointed at apex; dorsal branch rather narrow, slightly expanded apically, rounded at apex. Median lobe (Fig. 7K) long, slender, apical part narrow or slightly expanded. Phallobase with short tubular process (approximately 1/6 of total length) and long, slender furca.

Female genitalia: sternite VIII (Fig. 7F) with slightly bilobed apical protuberance, long setae situated at apex and alongside lateral margins; spiculum ventrale short, broadly clavate; St8L/St8W ratio: 1.62 (n = 1).

##### Sexual dimorphism.

Females are more robust, with protibiae not expanded and without fringe of long setae in basal part. Maxillary palpomere II not expanded in females and without long setae on ventral side. Terminal maxillary palpomere is wider in males, with its inner angle situated approximately in the middle (Fig. 7C), in females it is generally slenderer, with its inner angle situated in terminal 1/3 (Fig. 7D). Antennae are shorter in females; antennomeres V–X ~1.60× as long as wide in males, ~1.30× in females.

##### Distribution.

Croatia, France, Greece, Italy, Montenegro, Morocco, Spain. *Mordellistenapurpurascens* is reported here for the first time from Greece and Montenegro. Odnosum (2003, 2005, 2010) reported *M.purpurascens* from Kazakhstan, Turkmenistan, Tajikistan, and Ukraine. However, based on the figures of parameres provided in all the three mentioned studies, it is obvious that he referred to a different species (see Discussion).

##### Biology.

Adults were collected by the first author in Montenegro, in urban environment of Bar on the flowers of *Daucus* sp. (Apiaceae).

##### Remarks.

*Mordellistenapurpurascens* was described by Costa (1854) and referred to be found in several localities in former “Regno di Napoli” (southern parts of present Italy). Series of *M.purpurascens* in Costa’s collection in MZFN contains only two specimens. One of them with the original label “Mordellistenapurpurascens n. Napoli” is designed here as a lectotype. The other specimen labelled “S. Severina” without identification label belongs to a different species from the *gemellata*-group (sensu Ermisch 1956). Genitalia of the lectotype were examined for the first time for the purposes of the present study (Fig. 7G).

Ermisch (1977) briefly described two new species *M.geronensis* and *M.istrica* as a part of an identification key. He differentiated these species from each other based on the shape of the apical part of the median lobe (expanded in *M.istrica*, not expanded in *M.geronensis*). Shape of the apical part of median lobe depends on the observation method (dry/wet, card mounted/slide mounted). After examining the series of slide mounted median lobes of both taxa, we did not find any differences in the shape. Examination of the male genitalia from type specimens of *M.purpurascens* (Fig. 7G), *M.geronensis* (Fig. 7H) and *M.istrica* (Fig. 7I) revealed that these taxa are conspecific. We thus propose *M.istrica* and *M.geronensis* as the junior synonyms of *M.purpurascens*.

Type series of *M.istrica* includes a female paratype (Pola, Croatia), which we were not able to assign to *M.purpurascens* or *M.pseudohirtipes*. Type series of *M.geronensis* includes a male paratype (Bois de Paiolive, Ardêche, France), which in fact belongs to *M.pseudohirtipes*, and three paratypes (Tossa de mar, Spain; Costa Brava, Spain), which belong to *M.hirtipes*. In Ermisch’s collection, there is a series of specimens named *Mordellistenalopezi*. Such species has not been described. Specimen labelled as “Typus”, in fact, belongs to *M.purpurascens*, the rest of the specimens belongs to *M.pseudohirtipes*.

#### Mordellistena (s. str.) balearica

Taxon classificationAnimaliaColeopteraMordellidae

Compte, 1985

Fig. 8

Mordellistena balearica Compte, 1985: 63–64 (original description); Horák 2008: 96 (distribution).

##### Type locality.

Palma de Mallorca, Majorca.

##### Type depository.

According to the original description (Compte 1985), holotype should be deposited in MNCN. However, despite of the effort of the curator, the specimen was not found.

##### Diagnosis.

**Figures 8–9.**
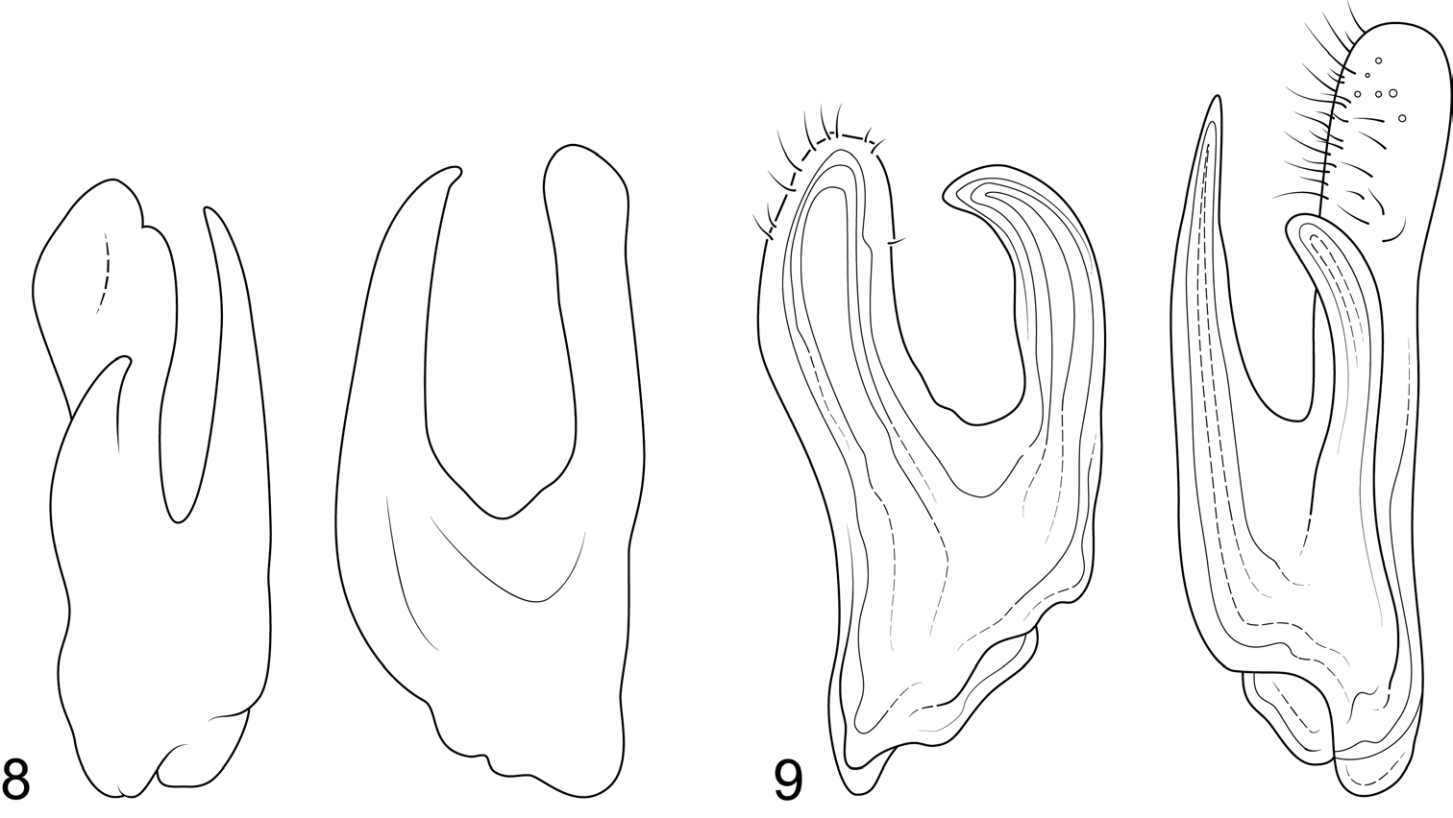
**8***Mordellistenabalearica* Compte, 1985, parameres (Compte 1985, modified) **9***M.irritans* Franciscolo, 1991, parameres (Franciscolo 1991, modified).

*Mordellistenabalearica* was described based on a single male specimen from Mallorca. According to the original description, this species closely resembles *M.pseudohirtipes* and can be distinguished from this species by longer antennae (antennomeres V–X two times longer than wide) and different shape of parameres (Fig. 8) (Compte 1985). All characters mentioned in the original description suggest that this taxon is conspecific with *M.pseudohirtipes*; unfortunately, the authors did not have the opportunity to study the type.

##### Distribution.

Known only from type locality.

##### Remarks.

Compte (1985) mentioned following information: “This specimen, together with other specimens collected by P. López in Majorca, which current location I don’t know, was studied by the specialist Mr Ermisch, who considered it as a new species for science, called *M.balearica*, a name that seems to have remained *in litteris*”. There are several specimens collected by P López in Mallorca in Ermisch’s collection (SNSD) labelled by Ermisch as *M.balearica* which in fact all belong to *M.thuringiaca* Ermisch, 1963.

#### Mordellistena (s. str.) irritans

Taxon classificationAnimaliaColeopteraMordellidae

Franciscolo, 1991

Fig. 9

Mordellistena irritans Franciscolo, 1991: 168–173 (original description); Franciscolo 1995: 12 (distribution); Horák 2008: 98 (distribution).

##### Type locality.

Lampedusa Is., Italy.

##### Type depository.

Museo d’Aumale, Terrasini, Palermo, Italy: 1 ♀ holotype, 1 ♂ paratype (Franciscolo 1991). Not examined.

##### Diagnosis.

*Mordellistenairritans* can be assigned to *M.hirtipes* complex based on the expanded maxillary palpomere II in males and the shape of parameres. This species can be distinguished from all other species in the complex by the characteristic shape of the left paramere with dorsal branch parallel-sided and rounded at apex (Fig. 9).

##### Distribution.

Known only from the type locality.

### Key to the species of *M.hirtipes* species complex

**Table:** 

1	Dorsal branch of the left paramere not expanded, parallel-sided, rounded at apex (Fig. 9)	*** M. irritans ***
–	Dorsal branch of left paramere expanded apically, obliquely truncate at apex	**2**
2	Parameres shorter, EL/LPrL ratio: 7.87–9.17 (8.48 ± 0.40, n = 14); EL/RPrL ratio: 10.07–11.89 (11.10 ± 0.50, n = 14); basal part of left paramere short; ventral branch of the right paramere usually distinctly shorter than the dorsal one (Fig. 5D–G). Terminal segment of maxillary palpi in females shorter and broader, inner angle is more acute (Fig. 4B). Pubescence on pronotum and elytra yellowish, somewhat darkened posteriorly	*** M. hirtipes ***
–	Parameres longer, EL/LPrL ratio: 4.42–7.17, EL/RPrL ratio: 5.57–8.63; basal part of left paramere longer; ventral branch of right paramere equally long or longer than the right one (Figs 6E–H, 7G–J). Terminal segment of maxillary palpi in females slenderer, its inner angle is rounded (Figs 6D, 7D). Pubescence on pronotum and elytra sometimes completely dark greyish	**3**
3	Parameres shorter, EL/LPrL ratio: 4.65–7.17 (5.89 ± 0.71, n = 25), EL/RPrL ratio: 5.91–8.63 (7.42 ± 0.72, n = 25); basal part of left paramere shorter (Fig. 6E–H). Pubescence on pronotum and elytra sometimes almost completely dark greyish	*** M. pseudohirtipes ***
–	Parameres longer, EL/LPrL ratio: 4.42–5.84 (4.98 ± 0.35, n = 19), EL/RPrL ratio: 5.57–6.94 (6.19 ± 0.41 n = 19); basal part of left paramere longer (Fig. 7G–J). Pubescence on pronotum and elytra yellowish, darkened posteriorly but not completely dark greyish	*** M. purpurascens ***

## Results of PCA analysis

Principal Component Analysis (PCA) was conducted based on following morphometric characters: HL, HW, PL, PW, EL, EW, PTiL, MsTiL, MtTiL, RPrL, BRPr, and LPrL. Characters were measured in 59 male specimens, including holotypes / lectotype of every taxon. The first two principal components describe 91.03% (PC 1) and 6.32% (PC 2) of variation. PC 1 correlates mostly with elytral length (loading 0.66), elytral width (loading 0.37) and pronotal width (loading 0.35); PC 2 correlates with characters measured on parameres: left paramere length (loading 0.68), right paramere length (loading 0.56), basal part of left paramere length (loading 0.43).

**Figure 10.**
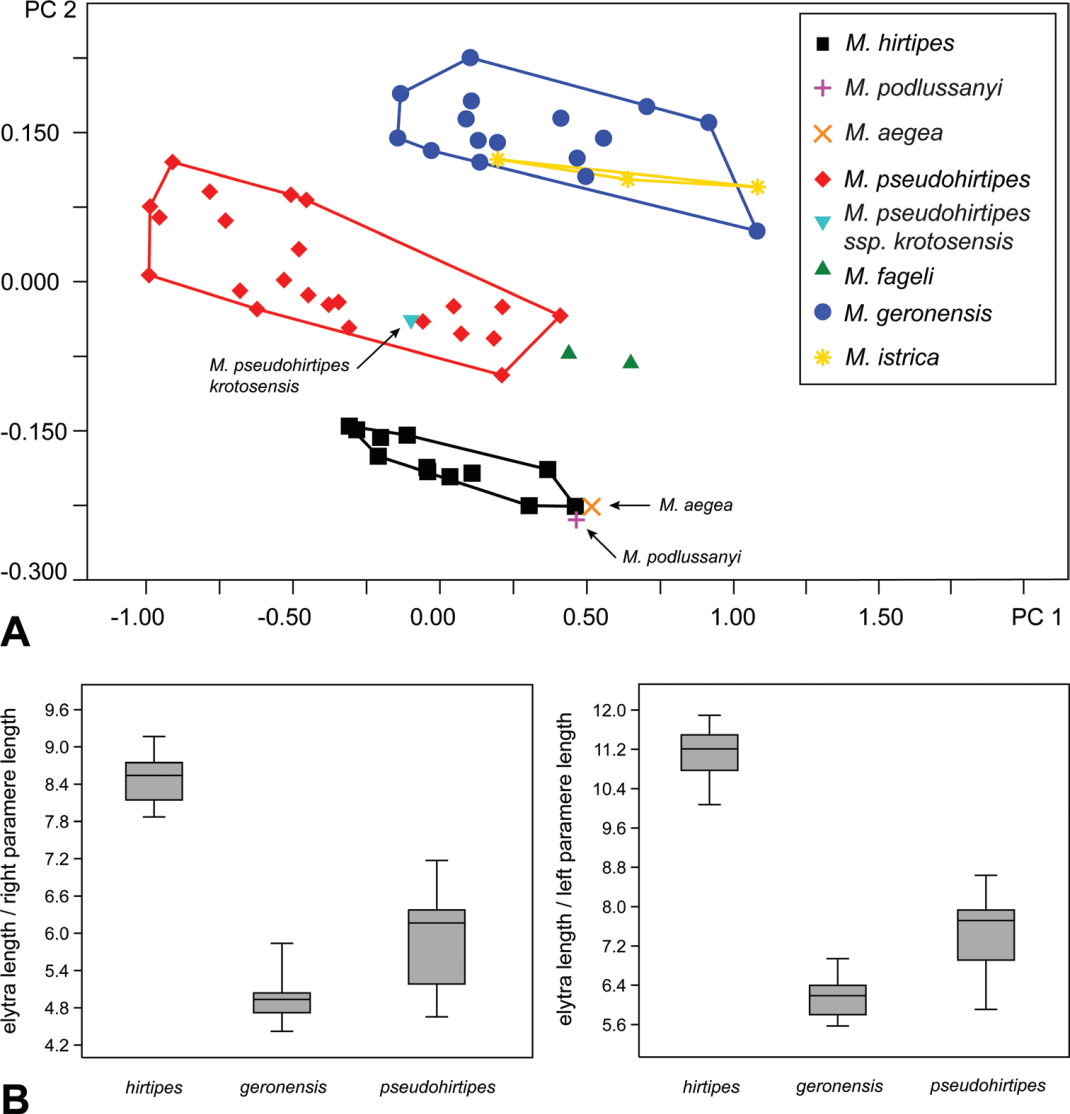
**A** Results of Principal Component Analysis (PCA) **B** Box-plots showing differences between species in selected ratios. Top and bottom of the boxes represent first and third quartiles, transverse band represents the median and whiskers represent maximum and minimum.

Visualisation of the results of PCA analysis (Fig. 10A) shows clear distinction of *M.hirtipes*, *M.pseudohirtipes*, and *M.purpurascens* along the PC 2 axis. Cluster of *M.istrica* overlaps with cluster of *M.geronensis*; *M.pseudohirtipeskrotosensis* is placed within the cluster of *M.pseudohirtipespseudohirtipes*; *M.fageli* is placed next to the cluster of *M.pseudohirtipes* in one plane along the PC 1 axis, and *M.podlussanyi* and *M.aegea* are placed next to cluster of *M.hirtipes*. Results of PCA correspond with hypotheses based on observations of morphological characters.

Length of elytra and length of parameres are characters, that reach the highest loadings in PCA analyses. Ratios of these characters (EL/RPrL, EL/LPrL) are useful for identification and are used in diagnoses. Differences in values of selected ratios are presented in Fig. 10B.

## Discussion

The family Mordellidae is taxonomically very challenging and thus rather poorly known. Most of the original descriptions are insufficient for proper identification and differentiation of the species, especially those published before the 1950s (before K Ermisch provided a more precise method of description). There are still some species which were described as several different taxa, sometimes even by the same author (e.g., *M.pseudohirtipes* Ermisch, 1965 = *M.fageli* Ermisch, 1969). Characters used for the differentiation of these taxa were usually misinterpreted (e.g., the shape of the median lobe in *M.geronensis* Ermisch, 1977 and *M.istrica* Ermisch, 1977) or they are subjects of the intraspecific variability (e.g., the dark coloration of the pubescence in *M.fageli* Ermisch, 1969). In other cases, the insufficient descriptions in combination with overlooking of the type specimens led to a misinterpretation of the taxa. It can be seen for example in some species described by Achille Costa (1854). Revision of the type specimens in his collection deposited in MZFN revealed that several species described by him were incorrectly interpreted by the subsequent authors as completely different species, one of them is *M.purpurascens* Costa, 1854 treated in the present paper. This species was considered by the subsequent authors as a synonym of either *M.pumila* (Gyllenhal, 1810) (Gemminger and Harold 1870) or *M.micans* (Germar, 1817) (Emery 1876; Baudi di Selve 1877; Heyden et al. 1883, 1906; Schilsky 1898; Csiki 1915). Later it was treated again as a valid species by Ermisch (1977), Ermisch in Kaszab (1979) and Batten (1977) but none of these authors had studied the types and it is obvious, based on their figures of the genitalia that the specimens considered by them as *M.purpurascens* belong to a different species. Their misinterpretations were later followed by Odnosum (2003, 2005, 2010) who published several new distribution records for *M.purpurascens* which were then included in the catalogue by Horák (2008). Only the examination of the lectotype of *M.purpurascens* Costa, 1854 done by the first author revealed that it is conspecific with the types of *M.geronensis* Ermisch, 1977 and *M.istrica* Ermisch, 1977. As it can be seen from this example, examination and redescriptions of the type specimens are essential for the future studies, especially in such taxonomically difficult and species-rich family as Mordellidae.

We live in the era of the global biodiversity crisis caused by the anthropogenic interventions in the natural ecosystems. But how does these changes affect the diversity and distribution patterns of Mordellidae beetles is not known. Despite of the great effort of the authors such as Ermisch (e.g., 1956, 1965, 1969a), Horák (e.g., 1990, 2008) and Odnosum (e.g., 2003, 2005, 2010) who have summarised and published a vast number of distributional records, the information about distribution and ecology of many Palearctic species is still very poor and several species are reported only from a single locality stated in the original description. It is very important to gather and provide new distributional and ecological records, however, it is also essential to pay effort to correct identification of the specimens to guarantee the accuracy of the published biological data.

## Supplementary Material

XML Treatment for Mordellistena hirtipes

XML Treatment for Mordellistena (s. str.) hirtipes

XML Treatment for Mordellistena (s. str.) pseudohirtipes

XML Treatment for Mordellistena (s. str.) purpurascens

XML Treatment for Mordellistena (s. str.) balearica

XML Treatment for Mordellistena (s. str.) irritans

## References

[B1] Batten R (1976) Mordellidae (Coleoptera) from the South of France and Pyrenees. Entomologische Berichten 36: 164–171.

[B2] Batten R (1977) Two new Mordellidae (Coleoptera) from Southern Europe, and a key to the *Mordellistenamicans* group. Entomologische Berichten 37: 167–176.

[B3] Baudi di Selve F (1877) Coleotteri eteromeri esistenti nelle collezioni del R. Museo zoologico di Torino ed in altre italiane. Eteromeri delle famiglie susseguenti a quella dei tenebrioniti nei limiti della fauna europaea e circummediterranea. Atti della Reale Accademia delle Scienze di Torino 13: 765–866, 1027–1183.

[B4] Compte A (1985) Mordellidae de las islas Baleares (Coleópteros). Boletim da Sociedade Portuguesa de Entomologia 3 (Suppl. 1): 57–66.

[B5] Costa A (1854) Parte l a. Coleotteri, Eteromeri. Famiglia de’Mordellidei — Mordellidea. In: Fauna del regno di Napoli ossia enumerazione de tutti gli animali che abitano le diverse regioni di questo regno e la acque che la bagnano contenente la descrizione de’nuovi o poco esattamente conosciuti noc figure ricavete da originali vivente e dipinte al naturale. Gaetano Sautto, Naples, 32 pp. [plates XX–XXII]

[B6] Csiki E (1915) Mordellidae. In: Junk W, Schenkling S (Eds) Coleopterorum Catalogus. Pars 63. W. Junk, Berlin, 84 pp.

[B7] Czető Z (1990) New Mordellidae (Coleoptera) from the Mediterranean Region, and a key to the genus *Mordellistenula* Sthegolewa-Barowskaya. Folia Entomologica Hungarica 51: 25–30.

[B8] Emery MC (1876) Essai monographique sur les Mordellides de l’Europe et des contrées limitrophes. L’Abeille: Journal d’Entomologie 14: 1–128.

[B9] Ermisch K (1956) Mordellidae. In: Horion A (Ed.) Faunistik der mitteleuropäischen Käfer.Band V: Heteromera. Entomologische Arbeiten aus dem Museum G. Frey, Tutzing bei München, 269–329.

[B10] Ermisch K (1963) Die Mordelliden der Insel Cypren (Coleoptera, Heteromera, Mordellidae). Notuale Entomologicae 43: 49–67.

[B11] Ermisch K (1965) Neue Mordelliden von der Balkanhalbinsel (Coleoptera, Mordellidae). Reichenbachia 5: 251–272.

[B12] Ermisch K (1969a) Neue Mordelliden aus Europa, Nordafrika und dem Nahen Osten (Coleoptera, Mordellidae). Entomologische Blätter 65: 104–115.

[B13] Ermisch K (1969b) 79. Familie: Mordellidae. In: Freude H Harde KW Lohse GA (Eds) Die Käfer Mitteleuropas.Band 8. Teredilia, Heteromera, Lamellicornia. Goecke & Evers, Krefeld, 160–196.

[B14] Ermisch K (1977) Die *Mordellistena*-Arten Ungarns und benachbarter Gebeite sowie Beschreibung einer neuen *Hoshihananomia*-Art aus Siebenbürgen (Coleoptera, Mordellidae). Folia Entomologica Hungarica (Series Nova) 30: 151–177.

[B15] Franciscolo ME (1949) XIII° contributo alla conoscenza dei Mordellidi (Coleoptera: Heteromera). Memorie della Società Entomologica Italiana 28: 81–95.

[B16] Franciscolo ME (1991) Su alcuni Mordellidi e Scraptiidi (Coleopetra – Heteromera) delle Isole Pelagie. Naturalista Siciliano, Series 4, 15: 167–178.

[B17] Franciscolo ME (1995) Famm. Mordellidae. In: Angelini F, Audisio P, Bologna MA, De Biase A, Franciscolo ME, Nardi G, Ratti E, Zampetti MF, ColeopteraPolyphaga XII (Heteromera escl. Lagriidae, Alleculidae, Tenebrionidae). In: Minelli A Ruffo S La Posta A (Eds) Checklist delle specie della fauna italiana.Ed. Calderini, Bologna, 11–13.

[B18] Gemminger M Harold B de (1870) Familia LVIII. Mordellidae In : Catalogus coleopterorum hucusque descriptorum synonymicus et systematicus. Tom VII. Tenebrionidae, Nilionidae, Pythidae, Melandryidae, Lagriidae, Pedilidae, Anthicidae, Pyrochroidae, Mordellidae, Rhipidophoridae, Cantharidae, Oedemeridae. EH Gummi, München, 2105–2117.

[B19] Hammer Ø Harper DAT Ryan PD (2001) PAST: Paleontological Statistics software package for education and data analysis. Palaeontologia Electronica: 4, 1–9. http://palaeo-electronica.org/2001_1/past/issue1_01.htm.

[B20] Heyden L von Reitter E Weise J (1883) Catalogus coleopterorum Europae et Caucasi. Editio tertia. Libraria Nicolai, Berlin, 228 pp 10.1002/mmnd.48018830225

[B21] Heyden L von Reitter E Weise J (1906) Catalogus coleopterorum Europae, Caucasi et Armeniae Rossicae. Editio secunda. Friedländer & Sohn, Berlin, 774 pp.

[B22] Horák J (1983) Revision der *Mordellistena*-Arten aus der *pentas*-Gruppe (Coleoptera, Mordellidae). Entomologische Abhandlungen 47: 1–13.

[B23] Horák J (1990) Typenrevision einiger wenig bekanntner Arten aus der Gattung *Mordellistena* Costa (Insecta, Coleoptera, Mordellidae). Entomologische Abhandlungen 53: 125–142.

[B24] Horák J (2008) Mordellidae. In: Löbl I Smetana A (Eds) Catalogue of Palaearctic Coleoptera.Vol. 5. Tenebrionoidea. Apollo Books, Stenstrup, 87–105.

[B25] Horák J (2011) The world genera system of family Tumbling Flower Beetles (Coleoptera: Mordellidae) and its distribution in the Czech Republic. Unpublished Bachelor Thesis, Czech University of Life Sciences, Prague.

[B26] ICZN (1999) International Code of Zoological Nomenclature (4^th^ edn). International Trust for Zoological Nomenclature, London, 306 pp 10.5962/bhl.title.50608

[B27] Kaszab Z (1979) Felemás lábfejízes bogarak II. Heteromera II. (Mordellidae), Fauna Hungariae, 134. Akadémiai Kiadó, Budapest, 100 pp.

[B28] Kucharczyk H Kucharczyk M Stanisławek K Fedor P (2012) Application of PCA in Taxonomy Research – Thrips (Insecta, Thysanoptera) as a Model Group. In: Sanguansat P (Ed.) Principal Component Analysis – Multidisciplinary Applications.InTech, Rijeka, 111–126. 10.5772/2694

[B29] Lawrence JF Ślipiński A (2010) Mordellidae Latreille, 1802. In: Leschen RAB Beutel RG Lawrence JF (Eds) Handbook of Zoology, Coleoptera, Beetles, Morphology and Systematics (Elateroidea, Bostrichiformia, Cucujiformia partim), Vol.2. Walter de Gruyter, Berlin-New York, 533–537. 10.1515/9783110911213.533

[B30] Lu W Jackman JA Johnson PW (1997) Male genitalia and phylogenetic relationships in North American Mordellidae (Coleoptera). Annals of the Entomological Society of America 90: 742–767. 10.1093/aesa/90.6.742

[B31] Odnosum VK (2003) Mordellid beetles (Coleoptera, Mordellidae) in the fauna of Kazakhstan and Middle Asia. Communication 2. Vestnik Zoologii 37: 33–49.

[B32] Odnosum VK (2010) Zhuki gorbatki (Coleoptera, Mordellidae). Fauna Ukrainy. Volume 19. Part 9. Naukova Dumka, Kiev, 264 pp.

[B33] Plaza E (1983) Mordellidae (Col.) de la provincial de Madrid. Actas del I Congreso Ibérico de Entomologia 2: 567–577.

[B34] Przybycień M Wacławik B (2015) Morphometric measurements of *Bryodaemon* (Coleoptera: Curculionidae): contribution to phylogeny. Baltic Journal of Coleopterology 15: 129–136.

[B35] Ruzzier E (2013) Taxonomic and faunistic notes on Italian Mordellidae (ColeopteraTenebrionoidea) with redescription of *Falsopseudotomoxiaargyropleura* (Franciscolo, 1942) n. comb. Bollettino della Società Entomologica Italiana 145: 103–115. 10.4081/BollettinoSEI.2013.103

[B36] Ruzzier E Ghahari H Horák J (2017) A checklist of the Iranian Mordellidae (Coleoptera: Tenebrionoidea). Zootaxa 4320: 146–158. 10.11646/zootaxa.4320.1.8

[B37] Samin N Etebarian HR Sakenin H Ghahari H Mirutenko V (2016) A faunistic study of some Coleoptera families from Iran. Ukrainska Entomofaunistyka 7: 21–24.

[B38] Schaufuss C (1916) Calwer’s Käferbuch. Einfuhrung in die Kenntnis der Käfer Europas. Ed. 6, Vol. 2. E. Schweizerbart, Stuttgart, 709–1390.

[B39] Schilsky J (1895) Mordellidae. In: Küster HC Kraatz G (Eds) Die Käfer Europa’s.Nach der Natur beschrieben, Heft 31. Bauer & Raspe, Nürnberg, 1–100.

